# Intracellular mRNA phase separation induced by cationic polymers for tumor immunotherapy

**DOI:** 10.1186/s12951-022-01647-8

**Published:** 2022-10-08

**Authors:** Zhen Xing, Jing Xue, Xindian Ma, Congwei Han, Zhenzhen Wang, Shunhuang Luo, Chunming Wang, Lei Dong, Junfeng Zhang

**Affiliations:** 1 State Key Laboratory of Pharmaceutical Biotechnology, School of Life Sciences, Nanjing University, 163 Xianlin Avenue, Nanjing, 210093 China; 2 State Key Laboratory of Quality Research in Chinese Medicine, Institute of Chinese Medical Sciences, University of Macau, Taipa, 999078 Macau SAR China

**Keywords:** mRNA phase separation, Cationic polymers, TGFβ1, Tumor microenvironment, Immunotherapy

## Abstract

The formation of biomolecular condensates via liquid‒liquid phase separation (LLPS) is an advantageous strategy for cells to organize their subcellular compartments for diverse functions. Recent findings suggest that RNA or RNA-related LLPS techniques have potential for the development of new cellular regulation strategies. However, manipulating RNA LLPS in living cells has great challenges. Herein, we report that cationic polymers (CPs) have strong RNA LLPS-inducing activity. By introducing CPs into living cells or RNA solutions, significant RNA LLPS was verified through confocal imaging, turbidity assays, and fluorescence recovery after photobleaching (FRAP) tests. Among them, turbidity kinetics determinations indicated that the hydrophilic positively charged amino groups on the CPs play essential roles in RNA phase separation. Moreover, the LLPS induced by the cationic polymers dramatically changed the gene expression patterns in the cells. Interestingly, we found that TGFβ1 mRNA was highly encapsulated in the RNA droplets, which lowered the immunosuppressive capability of the tumor cells and triggered marked antitumor reactions in a mouse breast cancer model. Thus, we present here the CP-based modulation of RNA LLPS as a novel transcriptional manipulation method with potential for cancer immunotherapy drug development.

**Graphical Abstract:**

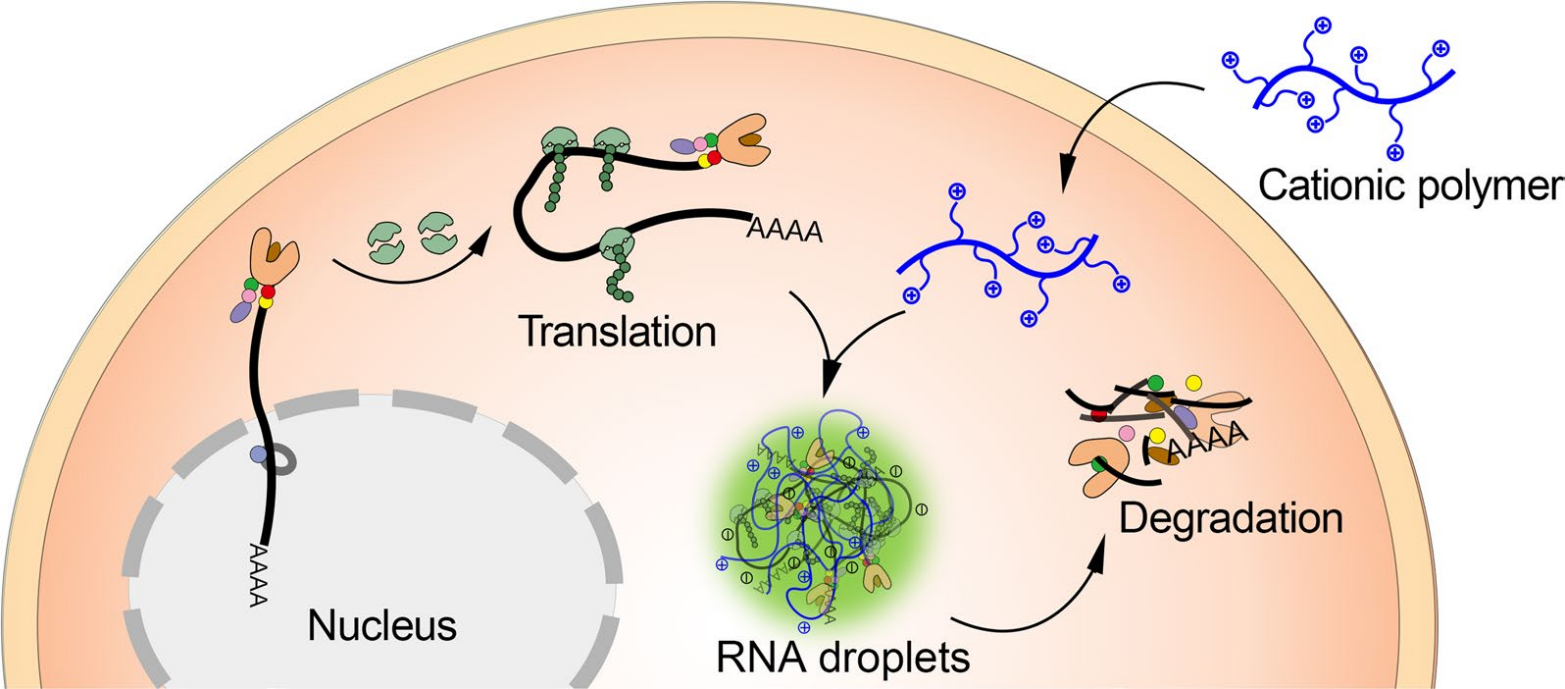

**Supplementary Information:**

The online version contains supplementary material available at 10.1186/s12951-022-01647-8.

## Introduction

Biomolecular condensation partitions cellular contents undergoing liquid–liquid phase separation (LLPS) and has important roles in signal transduction, stress responses, maintaining homeostasis [1], development [2], and disease [3, 4]. LLPS is a typical process in which macromolecules such as proteins or nucleic acids condense into a dense phase that often resembles liquid droplets, and this dense phase coexists with a dilute phase [5]. Notably, in recent cancer research, biomolecular condensates have been widely observed to directly regulate key cellular processes involved in cancer pathology, and the dysregulation of LLPS is increasingly implicated as a previously hidden driver of oncogenic activity [6]. Previous studies have mainly focused on protein-driven phase separation through multivalent interactions. RNA has generally been considered a regulatory component and scaffolding molecule in protein-driven phase separation [7]

Recently, it has become increasingly evident that many RNA-related cellular events involve LLPS, and many membraneless organelles have been found to contain RNAs [7]. RNAs undergo phase separation along with the corresponding RNA-binding proteins (RBPs) with binding RNAs as scaffolds [8]. Emerging studies have illustrated how RNA LLPS participates in RNA transcription, splicing, processing, quality control, translation, and function [9]. Studies have shown that RNA itself is also capable of phase separation, potentially through multivalent RNA–RNA interactions by forming RNA tandem repeat sequences [9] or RNA homopolymers [10]. Further evidence suggests that RNA LLPS has the capacity to influence gene expression [11]. Therefore, it is possible to develop new cell regulation strategies for RNA LLPS with specific active reagents.

As one of the most negatively charged macromolecules, RNA is prone to combination with materials with a positive surface charge, such as cationic nanoparticles [12]. This process has the potential to be used in the development of new strategies to induce desired cellular responses. However, the interaction between cationic particles and RNA is complex and difficult to control considering the corona formation process in the biological environment. The decoration of particles with a protein corona adsorbed from circulation or within the cytoplasm is inevitable, especially for particles with a cationic surface. This is an unpredictable process that alters the interaction between the particles with other molecules, including RNA. Cationic polymers, which have been used as gene-delivering carriers for decades [13], can package both DNA and RNA through the electrostatic interactions between the negatively charged phosphate backbone and positively charged residues of the polymer side chain. Therefore, we assume that cationic polymers would have strong interactions with intracellular RNA and drive RNA phase separation in living cells, which might alter the gene expression and the functions of the cell. As free mobile macromolecules, the combination of cationic polymers with other biomolecules is driven purely by the dynamics of the molecule/molecule affinity. The behavior of cationic polymers and their activities in biological environments are stable and would not suffer from the unpredictable modifications of the corona. This would make cationic polymer-induced RNA LLPS a potential way to develop new therapeutic strategies. To validate this, in the present study, we investigated three types of cationic polymers with the cytoplasmic condensates of RNA both in vitro and in vivo, with particular emphasis on the encapsulated mRNA in the process. We demonstrated that cationic polymers can arrest TGFβ1 mRNA translation by inducing RNA LLPS. These effects are able to reduce the immunosuppressive functions of the tumor microenvironment and enhance the efficiency of cancer immunotherapy.

## Results

### Intracellular mRNA phase separation induced by cationic polymers in vitro

**Fig. 1:**
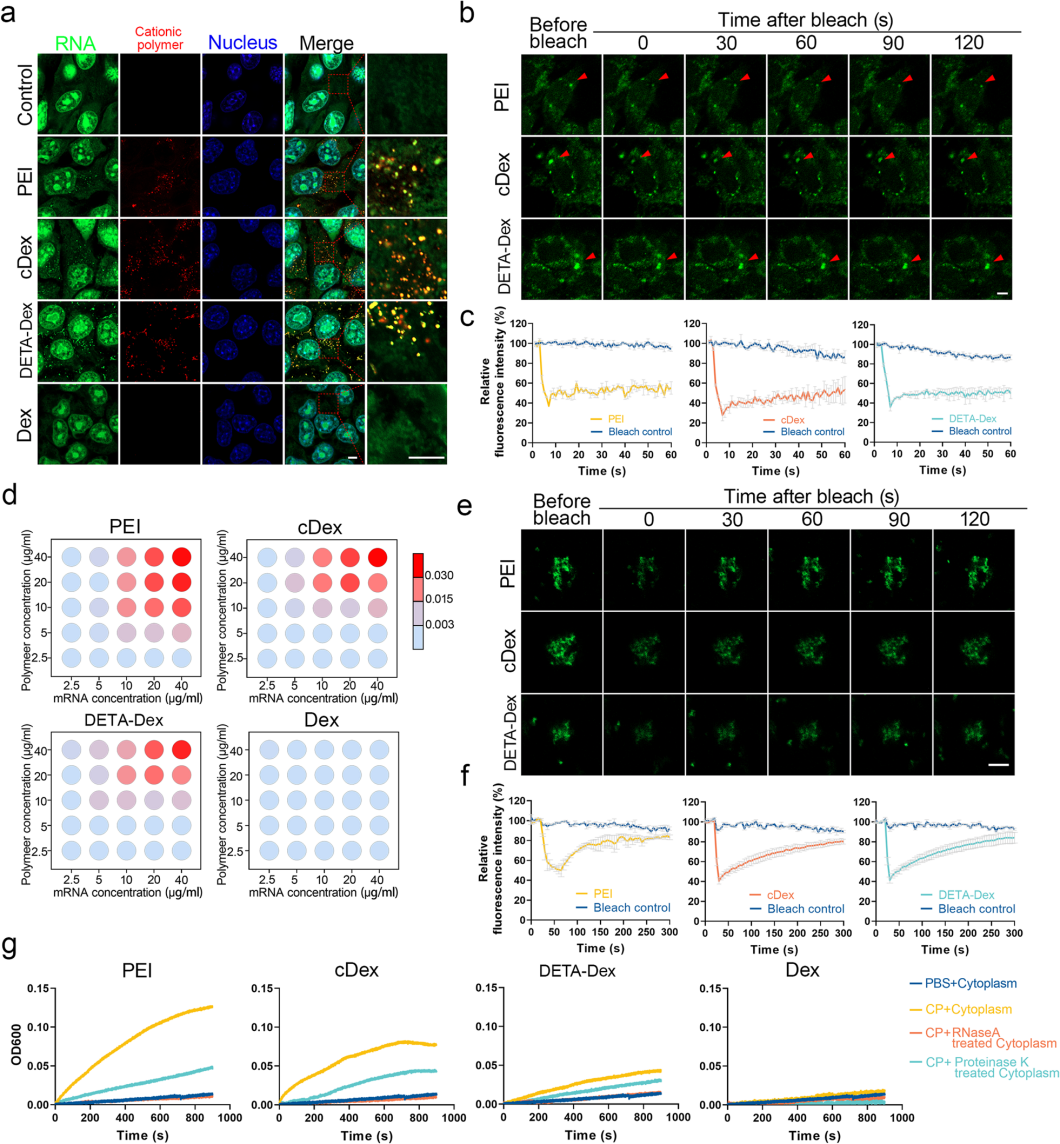
RNA LLPS induced by CPs. **a** Representative images of RNA droplets in 4T1 cells stained by SYTO RNAselect Green after treatment with different CPs. The CPs were labeled by Cy5. Scale bar, 5 μm. **b** Representative images of the FRAP experiment with Cy5-tagged CPs and SYTO RNAselect Green-stained RNA in 4T1 cells. Scale bar, 5 μm. **c** Quantification of the FRAP data (means ± SEMs, n = 3 experiments) of RNA droplets in 4T1 cells. **d** Phase diagrams of the CPs with concentrations ranging from 2.5 to 40 μg/ml in PBS (pH 7.0) and mRNA from 4T1 cells (ranging from 2.5 to 40 μg/ml). RNA was incubated with CPs in phase separation buffer at 37 °C for 30 min. Light dots, no phase separation; chromatic dots, phase separation. Phase separation was quantified by turbidity measurements at OD_600_. **e** Representative images of the FRAP experiment with Cy5-tagged CPs and SYTO RNAselect Green-tagged RNA in PBS solution. Scale bar, 5 μm. **f** Quantification analysis of the FRAP data (means ± SEMs, n = 3 experiments) in (**e**). **g** Kinetic analysis of the turbidity at OD_600_ in 4T1 cell cytoplasm with RNase or protease treatment before mixing with different CP solutions. 4T1 cell cytoplasm extract was treated with RNase A (20 μg/ml) at 37 °C and proteinase K (50 μg/ml) at 37 °C for 30 min

We first explored whether CPs could induce mRNA LLPS within living cells. In addition to PEI, which has been widely used in biological and medical studies, the other two cationic polymers (cDex and DETA-Dex) with completely different chemical structures (polysaccharides modified with polyamines) were synthesized and characterized accordingly (Additional file 1: Fig. S1, Tables S1 and S2). We chose appropriate concentrations of the different cationic polymers (PEI, cDex, and DETA-Dex) with relatively low cytotoxicity to treat living cells. When used to treat the cells, the polymers were labeled with Cy5 and displayed red fluorescence. The RNA aggregates were stained with SYTO RNAselect green fluorescent cell stain (Life Technologies). We monitored the cells for 48 h after the CP treatment. RNA staining revealed that many droplets formed in the cytoplasm of the cells immediately after CP treatment. The RNA droplets were strictly colocalized with the CP (Fig. 1a), indicating typical liquid‒liquid phase separation of the RNA together with the CP in the cytoplasm. Interestingly, the number of droplets gradually decreased after peaking at approximately four hours post-treatment (Additional file 1: Fig. S2a and S2b quantify the number of droplets per cell), suggesting a possible cellular clearing process. We performed fluorescence recovery after photobleaching (FRAP) experiments on these RNA droplets and found little RNA fluorescence recovery over 5 min, suggesting that the RNA in the droplets was immobile (Fig. 1b and c), which was different from most LLPSs of macromolecules in living cells [14]. Considering the possible influence of cytoplasmic proteins, we further evaluated the LLPS of RNA with CP using purified RNA from a mouse breast cancer cell line (4T1 cells) mixed with different CPs at various concentrations to establish a phase diagram (Fig. 1d). We found that the extent of RNA LLPS increased with increasing RNA and CP concentrations. As dextran without cationic modification did not trigger LLPS in the RNA solution, the cationic charge on the polymer molecules should be the key driving force for LLPS. This process was monitored with confocal microscopy (Additional file 1: Fig. S2c) and a digital camera (Additional file 1: Fig. S2d). We also performed FRAP experiments on the RNA droplets formed in the RNA/CP mixtures and found that in the part-droplet bleaching experiments, the bleached RNA signals efficiently recovered within 5 min, indicating that the unmixed RNA molecules readily interchanged with the RNA in the liquid-like droplets. This was different from the observations from the FRAP experiments performed in living cells (Fig. 1b), demonstrating that the RNA/CP droplets were in a solid-like state. The difference in these data might be due to the interaction between the initially formed RNA/CP droplets and some cytoplasmic proteins.

To measure the separation kinetics between the CP and RNA, we performed turbidity measurements (Additional file 1: Fig. S2e), and the interaction between the CP and RNA may be represented by the following equilibrium (1):

1 $$\left[CP\right]+\left[RNA\right]\rightleftarrows [CP/RNA]$$

which the extent of complex formation is determined by the forward and backward reaction rates. The forward rate constant (k_1_) was calculated according to the law of mass action [15] (2).

2 $${r}_{f}={k}_{1}\cdot {[CP]}^{\alpha }\cdot {[RNA]}^{\beta }$$

According to the results, the reaction rate of phase separation was positively associated with the cationic charge of the CP (Additional file 1: Fig. S2f).

We next assessed whether cytoplasmic proteins contributed to CP-induced LLPS in cytoplasmic extracts treated with RNase or protease. Treatment with RNase was found to have a greater influence on the generation of turbidity than protease (Fig. 1g), indicating that the CP had a preference for interacting with RNA than proteins and confirmed that the intracellular RNA LLPS was mainly due to the direct combination of the CP and RNA molecules. Altogether, these results demonstrated that CPs could trigger intracellular LLPS to form stable RNA droplets, which might sequester some mRNAs and downregulate their expression.

### RNA liquid‒liquid phase separation significantly downregulated TGFβ1 expression in the RNA droplets

**Fig. 2:**
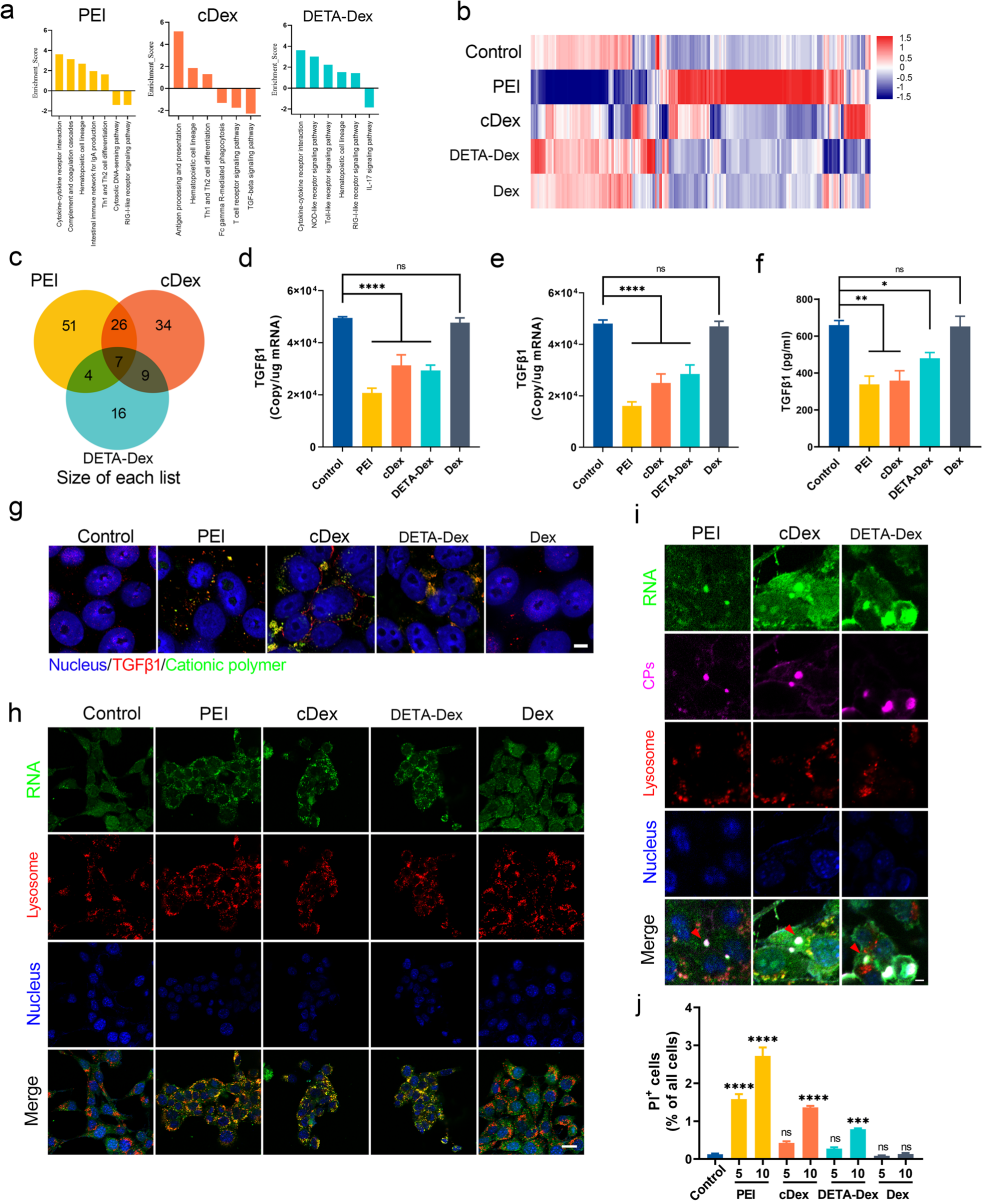
Cellular responses to RNA LLPS in CP-treated 4T1 cells. **a** Signaling pathways (KEGG) related to tumor immunity based on RNA-seq analysis of CP-treated 4T1 cells. **b** Heatmap of genome-wide RNA-seq profiling of 4T1 cells 24 h after CP treatment. **c** Venn diagram illustrating the number of tumor immunity-related genes significantly downregulated in CP-treated 4T1 cells. **d** Copy number of TGFβ1 mRNA transcripts in 4T1 cells after 6 h pf CP treatment and **e** 30 min of CP treatment. **f** ELISA measurements of TGFβ1 in CP-treated 4T1 cell culture medium. **g** Fluorescence in situ hybridization analysis showing TGFβ1 mRNA colocalization with the CPs in 4T1 cells. Scale bar, 5 μm. **h** Representative double-staining RNA droplet images with lysosomes in 4T1 cells after 4 h of CP treatment and **i** 15 min of CP treatment. Scale bar, 5 μm. **j** The signal intensity of PI fluorescence for 4T1 cells after incubation with cationic polymers (Polymer concentration, μg/ml). Data are expressed as the mean ± SEM, and the differences between experimental groups were analyzed by one-way ANOVA with Dunnett’s test (**d–f** and **j**). ∗*p* < 0.05, ∗∗*p* < 0.01, ∗∗∗*p* < 0.001, ∗∗∗∗*p* < 0.0001

To evaluate whether the CP-induced RNA LLPS could change gene expression in cells, we first performed RNA-seq on the cells treated with 5 μg/ml CP for 24 h. The sequencing data were analyzed using the Kyoto Encyclopedia of Genes and Genomes (KEGG) pathway database, and the signaling pathways enriched by at least two genes with fold enrichment values ≥ 2 were mapped (Fig. 2a and b). Notably, cell gene expression was significantly altered by CP treatment, and the main enriched pathways in our datasets were ‘cytokine‒cytokine receptor interactions’ and ‘antigen processing and presentation’ in both the PEI group and the DETA-Dex group. According to the Venn diagram analysis in Fig. 2C, we found seven common responsive genes in the three groups treated with different CPs (Additional file 1: Fig. S3a). After that, we used qPCR and ELISA to verify the alteration in expression of the seven genes. Based on the results (Fig. 2d–f and Additional file 1: Fig. S3b), TGFβ1 was the most significantly downregulated. We further showed that TGFβ1 mRNA was present in the CP-RNA droplets by using RNA fluorescence in situ hybridization (FISH) and RNA dot blot assays (Fig. 2f and Additional file 1: Fig. S3c). Interestingly, TGFβ1 mRNA was specifically captured by the LLPS droplets. One possible reason is that actively translating mRNAs such as TGFβ1 might be more easily accessed by cationic polymers as less is encapsulated by RNA-binding proteins [20]. To verify this, we selected ALP1 mRNA, with a similar abundance to TGFβ1 in the cytoplasm of 4T1 cells, as a control. We then evaluated their cellular protein contents by Western blot and detected a much higher protein level of TGFβ1 than ALP1 (Additional file 1: Fig. S3d), which meant TGFβ1 mRNA was more actively translated than ALP1. Meanwhile, the level of TGFβ1 and ALP1 mRNA in 4T1 cells by using QPCR (Additional file 1: Fig.S3e) and showing similar expression level. After that, by using dot blot assays, we evaluated the contents of the two kinds of mRNA in the LLPS droplets formed by PEI, cDex or DETA-Dex in the 4T1 cytoplasm. We found a higher level of TGFβ1 mRNA in these 3 different droplets (Additional file 1: Fig. S3f), suggesting the possible preferential binding of the polycations to the active transcripts.

To identify significantly enriched gene sets involved in 4T1 cells treated with each cationic polymers, gene set enrichment analysis (GSEA) was performed based on the mRNA microarray dataset (Additional file 1: Fig. S4). Significant enrichment of the ‘response_to_biotic_stimulus’ pathway with reduced expression of the edge gene subset in 4T1 cells was observed, and TGFβ1 was one of the downregulated genes in the gene set. Intriguingly, by using LysoTracker Red-labeled lysosomes, we observed that most of the RNA droplets had merged with the lysosomes 4 h and 30 min after their formation (Fig. 2g and i), which explained what had happened to the TGFβ1 mRNA after being encapsulated into the droplets during LLPS. We further filmed the process of the intracellular formation and degradation of the CP-RNA droplets with confocal microscopy (Additional file 2: Video S1, Additional file 3: Video S2 and Additional file 4: Video S3). Meanwhile, we observed that a large fraction of cationic polymers directly entered the cells rather than via endocytosis (Fig. 2i). To further verify this, propidium Iodide (PI) staining assay were used according to a previous literature method [16]. PI is readily internalized into cells with disrupted membranes but is excluded from cells with intact membranes. Fig.  2j showed the flow cytometer measurement of PI-positive cells that PI was internalized into the cells in a concentration dependent manner. Taken together, these data have shown that during CP-induced LLPS, the expression of TGFβ1 was significantly downregulated in 4T1 breast cancer cells. As TGFβ1 is one of the most effective immune suppressors during tumorigenesis and tumor development, it is possible that CPs could relieve immune suppression in the tumor microenvironment and activate antitumor immune reactions.

### CPs induced intratumorally RNA phase separation, TGFβ1 downregulation, and antitumour immune activation

**Fig. 3:**
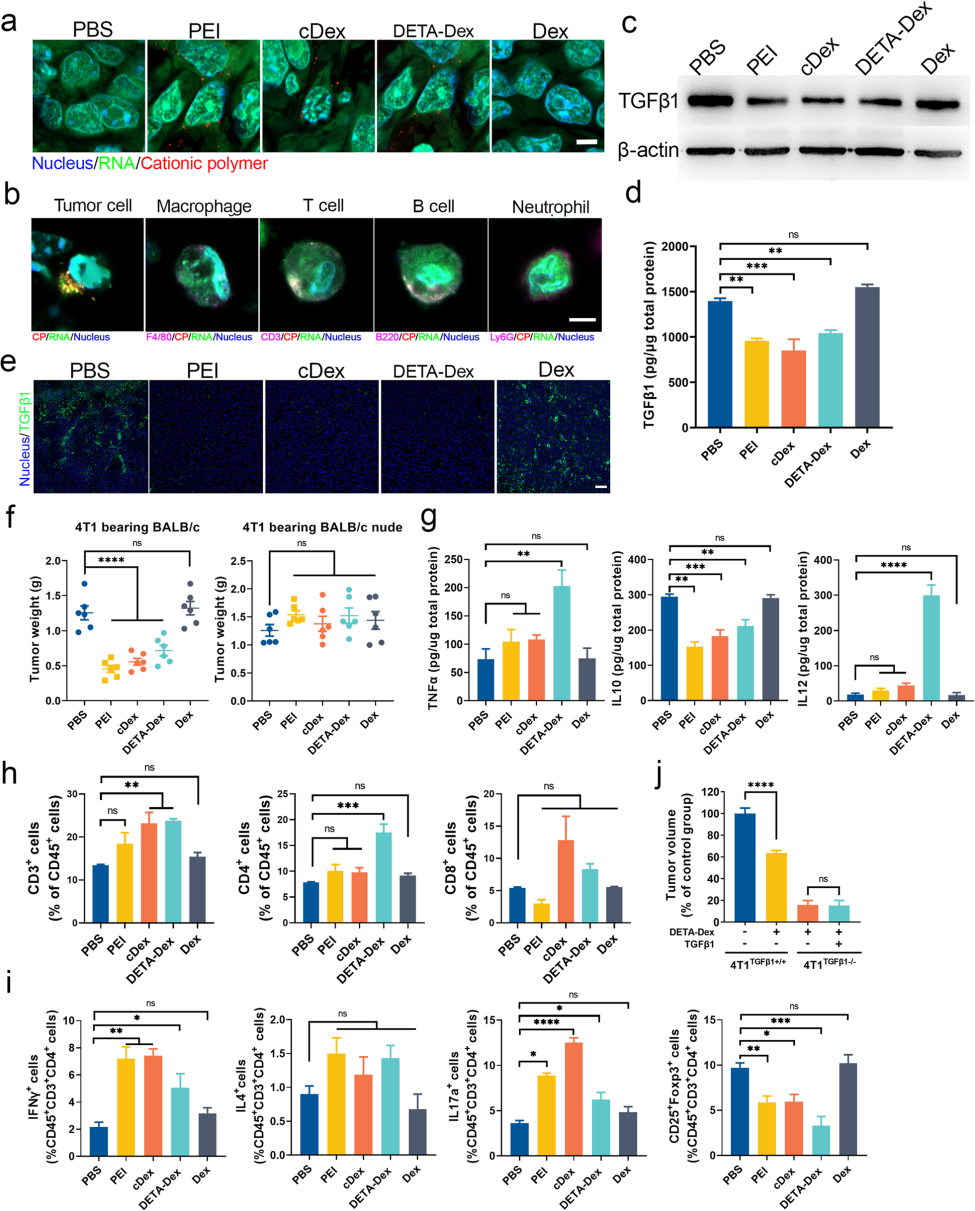
RNA LLPS, TGFβ1 downregulation, and antitumor immune activation in 4T1 tumor models after intratumoral CP administration. Mice bearing 4T1 tumors were intratumorally injected with the CPs (3 mg/kg body weight; dextran was used as a control reagent) 14 days before the mice were sacrificed, and the tissues were analyzed accordingly. **a** Representative confocal images of RNA droplets in tumor sections stained with SYTO RNAselect Green (scale bar, 5 μm). **b** Tumor cells and different leukocytes isolated from 4T1 tumor tissues were analyzed for RNA droplets marked by SYTO RNAselect Green (scale bar, 5 μm). **c** Western blot analysis of TGFβ1 in the tumor tissues after CP treatment. **d** ELISA analysis of TGF-β1 in tumor tissues after CP treatment. **e** Immunofluorescent staining of TGFβ1 in tumor tissue sections (scale bar, 50 μm). **f** Mean mouse tumor weights 14 days after the first CP treatment. **g** ELISAs of TNFα, IL10, and IL12; **h** the frequency of CD3^+^ T cells, CD4^+^ T cells, and CD8^+^ T cells; and **i** the frequency of Th1 cells, Th2 cells, Th17 cells, and Treg cells in the tumor tissues harvested from the same experiment as (**f**). **j** Evaluation of the antitumor activity of the CPs in the TGFβ1 knockout 4T1 cell animal model. Tumor size was normalized based on untreated 4T1^TGFβ1+/+^ cell-bearing mice, n = 10 per group. Data are expressed as the mean ± SEM, and the differences between experimental groups were analyzed by two-way ANOVA with Sidak’s multiple comparisons test (**i**) and one-way ANOVA with Dunnett’s test (**d**, **f**–**h** and **j**). ∗*p* < 0.05, ∗∗*p* < 0.01, ∗∗∗*p* < 0.001, ∗∗∗∗*p* < 0.0001

As cationic polymers were found to trigger antitumor immune responses in previous studies [17, 18], we performed in vivo experiments to examine whether CP-induced RNA LLPS and TGFβ1 downregulation played key roles. First, we tested whether CPs could induce RNA LLPS in vivo. CPs were administered via tail vein injection into model mice (BALB/c mice with transplanted 4T1 breast tumors). Two hours after injection, RNA droplets were observed in frozen tumor tissue sections from CP-treated mice (Fig. 3a). Additionally, we further subdivided the cells in the tumor tissues and found that the CP and RNA/CP droplets were mainly concentrated in the tumor cells rather than in the other types of cells in the tumor tissue (Fig. 3b). Second, treatment with all three different CPs significantly decreased TGFβ1 expression in the tumor tissue (Fig. 3c–e). Third, we tested the antitumor and immune activation effects of the CPs. CPs were given to the animals via intratumoral injection at a dosage of 3 mg/kg every 2 days. Fourteen days after the first administration, we evaluated the effects and found that BALB/c model mice treated with the CPs had significantly reduced tumor weights and sizes (Figs. 3f, Additional file 1: Fig. S5a, S5b and S5c). However, when treatment was performed in the same tumor model in BALB/c nude mice, there were no notable effects, suggesting that the antitumor effect of the CPs was dependent on the immune system. Therefore, we analyzed the levels of some key cytokines involved in the anticancer immune response and found that CP treatment markedly elevated the expression of tumor necrosis factor α (TNFα) and interleukin-12 (IL12) while lowering the expression of the immune suppressor interleukin-10 (IL10). (Fig. 3g). Consistently, CP treatment augmented the overall immune response, as evidenced by the elevated CD4^+^ and CD8^+^ T-cell levels in the tumor microenvironment (TME) (Fig. 3h). We further analyzed the production of IFN-γ, IL-4, IL17a, and Foxp3 in lymphocytes separated from the tumor tissues, which reflects the population of Th1, Th2, Th17, and Treg cells, respectively. According to the results (Fig. 3i), CP treatment increased IFN-γ and IL17a expression while reducing Foxp3 expression, suggesting that the CP-mediated TGFβ1 decrease promoted the Th1 and Th17 response but reduced the activity of immunosuppressive Treg cells. To verify the role of TGFβ1 in the CP-mediated antitumor effects, we constructed a 4T1 cell tumor mouse model with TGFβ1 knockout. As shown in Fig. 3j, the growth of the tumors with TGFβ1 knockout was significantly decreased compared with mice with normal 4T1 cells, and TGFβ1 protein supplementation was able to recover the growth capacity of the tumor. In the TGFβ1 knockout 4T1 model, CPs did not affect tumor growth. Together, these data suggested that the immune activation and antitumor effects of CP treatment were probably mediated by CP-triggered LLPS and TGFβ1 downregulation in the tumor cells.

### Tumor therapeutic effects of the CPs after intravenous administration

**Fig. 4:**
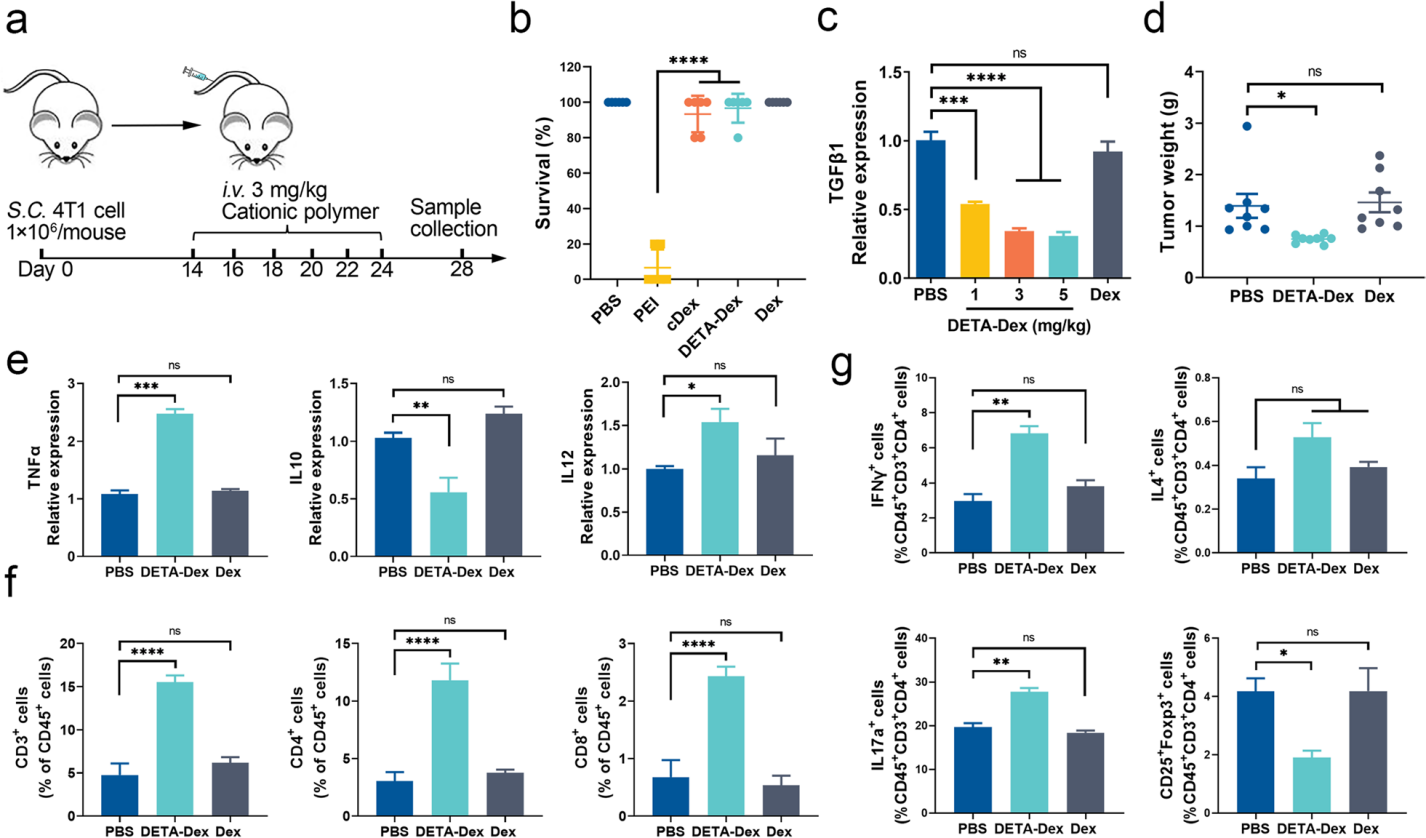
Antitumor activity of CPs after *i.v.* injection. **a** Schematic diagram of the experiment, n = 10 per group. **b** Acute toxicity test of the CPs. Different CPs were administered to the mice at 5 mg/kg via* i.v.* injection, and the survival rates were recorded 30 min after administration. The experiment was repeated three times, n = 5 per group. **c** The TGF-β1 mRNA level was measured by qPCR in the tumor tissues after DETA-Dex treatment. **d** Mean tumor weights in the model animals 14 days after the first DETA-Dex treatment (3 mg/kg body weight). **e** The mRNA levels of TNFα, IL10, and IL12, **f** the frequencies of CD3^+^ T cells, CD4^+^ T cells, and CD8^+^ T cells, and **g** the frequencies of Th1 cells, Th2 cells, Th17 cells, and Treg cells in the tumor tissues harvested from the same experiment in (**d**). Data are expressed as the mean ± SEM, and the differences between experimental groups were analyzed by two-way ANOVA with Sidak’s multiple comparisons test (**b**, **d**, **e**–**g**) and one-way ANOVA with Dunnett’s test (**c**). ∗p < 0.05, ∗∗*p* < 0.01, ∗∗∗*p* < 0.001, ∗∗∗∗*p* < 0.0001

As intratumoral drug administration is not common in clinical practice, we further tested the antitumor efficacy of the CPs after intravenous injection (Fig. 4a). First, the safety of the cationic polymers was assessed, as some of the cationic reagents have been reported to have toxicity, such as causing coagulation or hemolysis [19, 20] and found that DETA-Dex had the lowest toxicity (Fig. 4b). Therefore, we used DETA-Dex as the sole therapeutic to treat the model animals. In the therapy tests, the animals were given DETA-Dex (dosage of 1, 3, or 5 mg/kg) via the tail vein once every 2 days. Then, we analyzed the TGFβ1 contents in the tumor tissues. The results are shown in Fig. 4c, from which we chose the dose of 3 mg/kg as the optimized dose for subsequent experiments.

DETA-Dex therapy administered via* i.v.* injection significantly reduced the tumor weight (Fig. 4d). The T-cell responses after DETA-Dex therapy were also evaluated. Consistent with the results in previous intratumoral treatments, DETA-Dex enhanced the Th1 and Th17 responses while reducing Treg cell activity (Fig. 4e–g). Collectively, our data demonstrated that *i.v.* injection of DETA-Dex was feasible to treat tumors in vivo.

### The synergistic effect of DETA-Dex with PD1 therapy

**Fig. 5:**
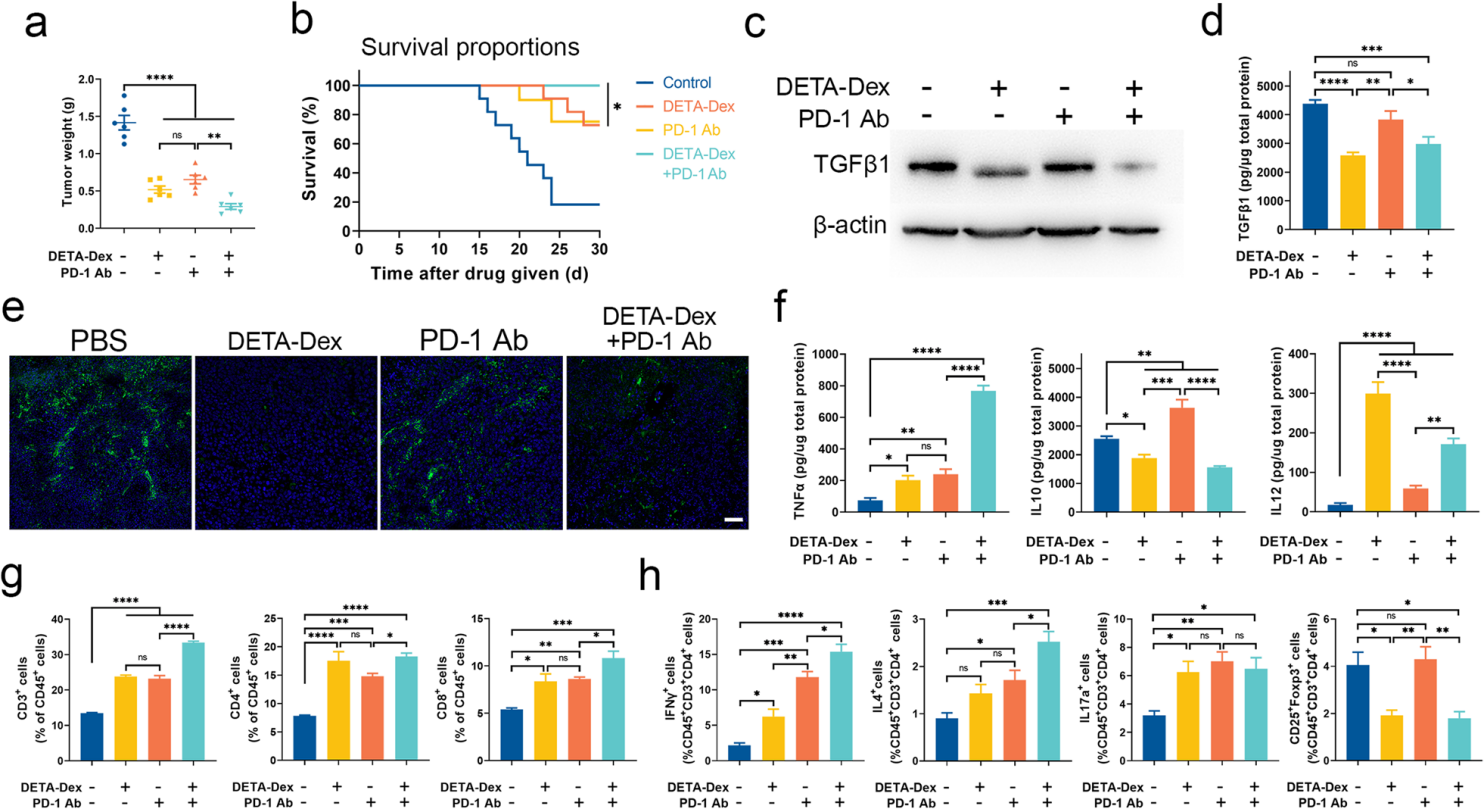
DETA-Dex enhanced PD-1 immunotherapy efficacy. DETA-Dex (3 mg/kg BW) and a PD-1 inhibitor (Bioxcell, #BE0146, 5 mg/kg BW) were intratumorally coinjected into 4T1 model mice every 2 days; each mouse received 6 injections before sacrifice. **a** Mean weights of the tumors harvested on Day 14 after the first treatment. **b** Survival of tumor-bearing mice after the indicated treatments, n = 10 per group. **c** Western blot analysis of TGFβ1 in tumor tissues; **d** ELISA of TGF-β1; **e** immunofluorescent staining of TGFβ1 in the tumor tissue sections (scale bar, 50 μm); **f** ELISAs of TNFα, IL10, IL12; **g** the frequencies of CD3^+^ T cells, CD4^+^ T cells and CD8^+^ T cells; and **h** the frequencies of Th1 cells, Th2 cells, Th17 cells and Treg cells in tumor tissues from the same experiment in (**a**). Data are expressed as the mean ± SEM, and the differences between experimental groups were analyzed by two-way ANOVA with Sidak’s multiple comparisons tests (**b**) and one-way ANOVA with Dunnett’s test (**a**, **d**, **f–h**); ∗*p* < 0.05, ∗∗*p* < 0.01, ∗∗∗*p* < 0.001, ∗∗∗∗*p* < 0.0001

TGFβ1 is a highly immunosuppressive cytokine that contributes to tumor immune escape by inhibiting antitumor T-cell activity [21], thereby reducing the efficiency and efficacy of immunotherapies that involve the activation of T cells. Therefore, we hypothesized that CP treatment would reduce TGFβ1 expression and enhance T-cell-mediated tumoricidal effects, improving the effectiveness of immune checkpoint therapies in cancer treatment. To prove this, we tested the effects of DETA-Dex treatment in combination with cancer immunotherapy. In the 4T1 breast cancer mouse model, a combination of DETA-Dex with anti-mouse PD-1 antibody significantly enhanced tumoricidal efficiency compared with treatments consisting solely of DETA-Dex or the PD-1 inhibitor (Fig. 5a, b, and Additional file 1: Fig. S7). We analyzed the TGFβ1 content in the tumor tissues (Fig. 5c–e) and found that the amount of TGFβ1 decreased in the DETA-Dex group and the DETA-Dex/PD-1 inhibitor combination group compared with the group treated with only the PD-1 inhibitor. Additionally, DETA-Dex treatment markedly elevated the expression of TNFα and IL12 while lowering the expression of interleukin-10 (IL10) (Fig. 5f). Moreover, further investigation of the tumor tissues demonstrated that DETA-Dex treatment markedly promoted T-cell-mediated antitumor immunity, as evidenced by the elevated levels of the cytokines TNFα and IL12, more CD4^+^ T cells infiltration into the tumor tissue (Fig. 5g and h), more Th1, Th2, and Th17 cells, and fewer Treg cells. These findings revealed the potential of the cationic polymer DETA-Dex to enhance the efficacy of conventional cancer immunotherapy.

## Discussion

In this study, we demonstrated that encapsulating mRNA transcripts via LLPS with cationic polymers could relieve tumor immunosuppression and inhibit tumor growth when administered alone or in combination with a PD-1 inhibitor. Our findings reveal for the first time that inducing RNA LLPS within tumor cells is a potential method for cancer therapy and anticancer drug development. Moreover, our study found unexpected specificity in mRNA silencing by CP-induced LLPS, although the mechanism remains to be elucidated. This phenomenon further highlights the translational potential of this strategy in cancer therapy.

LLPS has been intensively studied for its regulatory role in gene expression. Recent studies have shown that LLPS is essential for transcription factors, coactivators, and enhancers to regulate gene transcription [22–24]. For example, LLPS of the RNA polymerase Pol II and others initiates the formation of nuclear condensates and Pol II-mediated transcription [25]. A similar mechanism was also found during the formation of superenhancers that consist of a cluster of enhancers that activate the transcription of the key genes that determine cell identity [23]. Interestingly, alongside the LLPS of these transcription factors and coactivators, the transcribed RNAs also contribute to the formation of transcription condensates [11], indicating that RNA LLPS or RNA-mediated LLPS is an important method for transcription and cell activity regulation. Many studies have demonstrated that as one of the most abundant biomacromolecules in the cytoplasm, RNA is not only a constituent in LLPS condensates that are composed mainly of proteins, lipids, or DNA but also a regulator in some LLPS processes that are key for biological functions [26]. Additionally, RNA itself can self-separate [9]. For example, RNAs containing GGGGCC (rG4C2), CAG, and CUG repeats present typical LLPS in vitro or in living cells. G4-containing RNAs act as molecular scaffolds to sequester specific RBPs (such as G3BP1), thus regulating specific cellular processes [27, 28]. Therefore, RNA LLPS and RNA-participating LLPS can serve as unique new types of targets for the development of therapies and drugs.

RNA LLPS has several important advantages that make it a better candidate for the regulation of gene expression. First, the arrest of gene translation through induced RNA LLPS can be considered terminal interference, which should be more specific than inhibition at upstream checkpoints. Second, compared to siRNA or antisense oligonucleotides, RNA LLPS can be induced by reagents that do not need a special delivery system, which is the primary obstacle for nucleic acid drug development. Third, RNA LLPS represents a transient state of RNA molecules and is commonly reversible, which makes regulating cell activity occur more quickly and provides a more subtle way to treat certain special pathological conditions, such as neurological diseases.

CPs have been investigated as carriers for the delivery of nucleic acid drugs for years. Because they can strongly combine with DNA or RNA, CPs are ideal candidates for developing RNA LLPS inducers. In our study, two cationic dextran derivatives, cDex and DETA-Dex, were synthesized with comparable RNA LLPS-inducing effects. We demonstrated that the use of cDex and DETA-Dex had no observable adverse effects in mice. Compared with nondegradable PEI, the most investigated CP in drug delivery, cationic dextrans are degradable in the cytoplasm, which is important for the long-term safety of the treatment [29]. Our previous studies showed that PEI and other CPs can activate antitumor immunity through TLR signaling and repolarize MDSCs or macrophages into the immune-promoting phenotype [17, 18]. However, when applied in vivo, after entering into the tumor tissue, most of the CP molecules are captured by tumor cells, which was also evidenced in our present study. Therefore, the immune activation effects might also (at least partly) be derived from the reactions of the tumor cells to the CP molecules within the cytoplasm. As discussed above, the cationic property of CP molecules endows them with the capacity to combine with anionic macromolecules. Among all of the biomacromolecules in the cytoplasm, RNA has the strongest negative charge. From the point of combination kinetics, CP molecules will preferentially interact with RNA, which was demonstrated in our study, as CP/RNA combination occurred immediately after CP presented in the cytoplasm. This CP/RNA interaction is the event that triggers all subsequent cellular responses.

There were two interesting and unexpected findings in our present study. First, the CP/RNA droplets that formed in living cells were gel-like, which is different from those formed in solution, with typical liquid-like and more dynamic properties. The intracellular LLPS seemed to not be reversible, which is similar to disease condensate states resulting from an imbalance of RNA or protein components that lead to condensate ‘hardening’ [30]. Because CPs with a highly positive surface charge can also interact with some proteins in the cytoplasm, we predict that the RNA/protein ratio with CP-induced variation might cause transformation of the droplet properties. In our study, the gel-like condensates activated the autophagy‒lysosome pathway to degrade agglomerated RNA [31, 32] and strengthened the regulatory effects. Second, we found that the seemingly nonspecific combination of the CPs and RNA had significant specificity for the treatment of tumors. Tumor-promoting genes, including TGFβ1, were more likely to be packaged into LLPS droplets. We hypothesize that tumor cells are constantly expressing genes for proliferation and immune suppression [33], and the actively translating mRNA in the polyribosomes might facilitate their recognition by CPs and increase the likelihood of undergoing LLPS in the cytoplasm [34]. This is consistent with the finding that under stress conditions, mRNAs show dynamic movement between polysomes, P-bodies, and SGs [35]. However, systematic studies are needed to elucidate the detailed molecular events during RNA LLPS induced by CPs or other reagents to provide a complete mechanical explanation. Based on further mechanical discovery, it is possible to design CPs with more specific targets, which is of great significance for future drug development.

## Conclusion

In summary, we demonstrated a new strategy to regulate gene expression in tumor cells via electrically induced LLPS with synthetic CPs. In our present study, CP-induced intracellular LLPS caused dramatically reduced TGFβ1 expression in tumor cells in vitro and in vivo. Based on our data, this process was highly efficient in tackling immunosuppression and enhancing immunotherapy efficacy in a mouse model. Our findings highlight the unique potential of LLPS as a novel way to develop anticancer therapeutics with further investigations into the biological functions and mechanisms of inducible LLPS.

## Materials and methods

### Reagents

PEI (25 kDa) was purchased from Sigma (St. Louis, MO, USA). Dextran (70 kDa) and N,N-carbonyldiimidazole (CDI) were purchased from Aladdin Reagent Co., Ltd. (Shanghai, China). Other chemical reagents were purchased from Sangon Biotech (Shanghai, China) unless otherwise stated.

### Cationic polymer preparation

Cationic polymers were synthesized according to a previously reported method [17, 36]. Briefly, for cDex, 0.3 g of dextran dissolved in DMSO (30 ml) was incubated with 0.9 g of CDI for activation. Two hours later, 2.5 ml of anhydrous ethylenediamine was cautiously added dropwise for the cross-linking reaction. Then, the mixture was collected 24 h later and dialyzed against deionized water for 3 days. For DETA-Dex, the reaction was similar to that with cDex, only ethylenediamine was replaced with diethylenetriamine (DETA). After vacuum drying, the obtained cDex/DETA-Dex was kept in a dryer. The cationic polymers were characterized by ^1^H NMR (AVANCE III HD 400, Brook Corporation, USA), elemental analysis (Vario MICRO cube, Elementar, Germany), and Fourier transform infrared spectroscopy (NEXUS 870., NICOLET, USA) in the scanning range of 4000–400 cm^−1^.

To detect the degradability of the cationic polymers, cationic polymer (50 mg) was incubated with fresh mouse serum (5 ml) for 24 h. Then, the protein was removed by the Sevag method, and the cationic polymers were precipitated with acetone (PEI) or absolute ethanol (cDex and DETA-Dex). The cDex and DETA-Dex precipitates were dissolved in H_2_O (3 mg/ml) and passed through a 0.45 μm syringe filter for homogeneity and molecular weight analysis by the HPLC–RID method. The chromatographic conditions were as follows: an Agilent 1200 series LC system (Agilent Technologies, Palo Alto, CA, USA) was equipped with a TSK-GEL G4000PWXL column (300 mm × 7.8 mm, 10 μm) and a differential refractive index detector (RID; G1362A, Agilent Technologies, Palo Alto, CA, USA). During the experiment, the flow rate was maintained at 0.4 ml/min, and the column temperature was kept at 40 °C. For all separations, the mobile phase was ultra-pure H_2_O. Data acquisition and analysis were carried out using Astra software (version 6.0.2, Wyatt Technology Co., Santa Barbara, CA, USA).

### In vitro phase separation assay

Fluorescent staining of RNA was performed using a standard protocol. Briefly, 4T1 cells were grown on glass coverslips to 80% confluence and then treated with 10 μg/ml cationic polymer (PEI, cDex, and DETA-Dex) in RPMI-1640 for the indicated times (1 h, 2 h, 4 h, 6 h, or 48 h). Cells were washed three times with cold PBS and fixed with 70% ethanol. Next, the cells were washed twice, and the detection reagent (500 nM SYTO RNAselect Green and 5 μg/ml DAPI nuclear stain in 1 × PBS) was added for 30 min of incubation at RT in the dark, followed by an additional three washes. After staining and sealing, the cells were analyzed under a Zeiss LSM980 microscope with a Zeiss Plan-Apochromat 63 × /1.4 oil objective. The progress of phase separation in vitro was recorded with a digital camera. Briefly, 200 μg of 4T1 mRNA in 100 μl of H_2_O was placed in a 0.2 ml polypropylene PCR tube, 20 μg of cationic polymer was added, and videos were recorded with a digital camera. To study the kinetics of the phase transition, the OD value at 600 nm was measured using a UV‒Vis spectrophotometer (UV-2550, Shimadzu). For cytoplasm RNase or protease treatment, 4T1 cell cytoplasm extract was treated with RNase A (20 μg/ml) at 37 °C and proteinase K (50 μg/ml) at 37 °C for 30 min, and then the supernatants were separated by centrifugation (12,000 rpm, 4 °C for 20 min). Each concentration consisted of triplicate samples, and each experiment was performed at least twice. The protein, RNA, and DNA contents in the droplets in the cytoplasm of the 4T1 cells were determined with a BCA protein quantitation kit and an iQuant™ High Sensitivity Quantitation Kit (GeneCopoeia).

### In vitro and in vivo FRAP assays

For the in vitro RNA FRAP experiment, the droplets were photobleached with 100% laser power for 5 s using 488 nm lasers. Time-lapse images of the sample were collected after 100 ms of exposure time at 1 frame every 5 s using a Zeiss Plan-Apochromat 20 ×/1.0 water objective. Cellular FRAP was performed on a Zeiss LSM880 confocal microscope system at 37 °C in a live-cell imaging chamber. The RNA droplets were fully photobleached with 100% laser power for 2 s using a 488 nm laser. Time-lapse images were acquired over 5 min after bleaching. Images were processed by Zen Blue 3.1 software, and FRAP data were fitted to a single exponential model using GraphPad Prism 8.

### mRNA sequencing microarray analysis

4T1 cells treated with cationic polymer for 24 h were flash-frozen in liquid nitrogen and stored at − 80 °C for microarray expression analysis. Total RNA from the cells was isolated using TRIzol according to the manufacturer’s instructions, and RNA concentration and purity were measured using a NanoDrop ND-1000 spectrophotometer (NanoDrop Technologies Inc., USA). The integrity of the RNA was determined by denaturing agarose gel electrophoresis. RNA samples were further purified, converted into cDNA, and labeled according to the Agilent One-Color Microarray-Based Gene Expression Analysis protocol (Agilent Technology). After hybridization and washing, the chips were scanned with an Agilent DNA Microarray Scanner. Agilent Feature Extraction software (version 11.0.1.1) was used to analyze the acquired array images. Quantile normalization and subsequent data processing were performed using the GeneSpring GX v12.1 software package (Agilent Technologies). After quantile normalization of the raw data, genes for which at least 1 out of 5 samples with detected flags (“all targets value”) were chosen for further data analysis. Differentially expressed genes between the two samples were identified through fold change filtering. Hierarchical clustering was performed using R scripts. GO analysis and pathway analysis were performed using the standard enrichment computation method. To determine whether specific biological pathways were differentially affected by the cationic polymers, we analyzed our microarray dataset using the Kyoto Encyclopedia of Genes and Genomes (KEGG) pathway database (http://www.genome.jp/kegg/pathway.html). The Jvenn (http://jvenn.toulouse.inra.fr/app/index.html) online tool was used for cross-analysis to obtain lists of the exclusive and common genes among the cationic polymers [37].

### Dot blot hybridization

For RNA droplet preparation, 50 μg of 4T1 mRNA and 5 μg of cationic polymer were incubated for 30 min at 37 °C and then isolated by centrifugation at 15,000 rpm for 15 min. The precipitates were washed briefly three times with DEPC-treated ultrapure water and resuspended in 50 μl of water. For membrane preparation, the nylon membranes were soaked in 2 × SSC (1 × SSC comprising 0.15 M sodium chloride and 0.015 M sodium citrate, pH 7.0). Fifty microliters of RNA droplets were added to each dot with micropipette tips, and the membranes were dried for 30 min at room temperature and immobilized by UV exposure in a UV crosslinker. Then, the membranes were immersed in hybridization tubes with 10 ml of prehybridization solution preheated at 42 °C for 12 h in a shaking water bath. Portions (µg) of the probes were denatured in a boiling water bath for 10 min and then immediately placed in an ice bath for 5 min. The prehybridization solution was removed and replaced with 10 ml of hybridization solution containing 6 × SSC, 20% formamide, 5 × Denhardt’s solution, 0.5% SDS (w/v), 500 µg/ml yeast tRNA, and 30 ng/ml biotin-labeled DNA probes. The hybridization tubes were resealed and incubated for 3 h at 42 °C. The hybridization solution was then removed, and the membranes were washed briefly three times with washing buffer (2 × SSC-0.1% (w/v) SDS) for 10 min at room temperature. The membranes were then incubated in 10 ml of HRP-streptavidin (1:500) for 30 min at 42 °C and rinsed briefly three times with 10 ml of washing buffer for 15 min at room temperature. Enhanced ECL chemiluminescence detection solution (2 ml) was added to the tubes in the darkroom for analysis by scanning.

### Mouse model

BALB/c mice and BALB/c nude mice (female, 6 weeks of age; Vital River Laboratory Animal Technology Co. Ltd., Beijing China) were housed in a specific pathogen-free (SPF) animal facility with controlled light (12-h light/dark cycles), temperature, and humidity conditions, and were fed a standard chow diet with water available. All experimental procedures involving animals were approved by the Institutional Animal Care and Use Committee of Nanjing University.

The mouse mammary carcinoma cell line 4T1 was obtained from ATCC (CRL-2539). Cells were grown and maintained in RPMI-1640 medium supplemented with 10% FBS. To generate the heterotopic tumor model, 1 × 10^6^ cells were injected subcutaneously into the left armpits of the animals. We measured the tumor size with a Vernier caliper and weighed the tumor samples upon harvest. The tumor volumes were calculated as follows: tumor volume (mm^3^) = 0.5 × length × width × height. The antitumor activities of the cationic polymers were examined in heterotopic 4T1 tumor-bearing mice. When the tumor size reached approximately 0.5 cm in diameter, the tumor-bearing mice were injected with PBS, PEI, cDex, DETA-Dex, or Dex (3 mg/kg body weight) every 2 days. After drug administration, the tumor sizes were examined every 2 days. Fourteen days after the first administration of cationic polymer, all tumors were excised and weighed, and all tumor tissues were sectioned for histopathological and immunofluorescent analyses. Once the tumor size reached 1.3 cm, the study was terminated, and the mice were sacrificed. To evaluate the toxicity, healthy BALB/c mice were given the therapeutic agents via the caudal vein (5 mg/kg, *i.v.* injection), and the survival rate was recorded 30 min after administration.

To analyze the effect of the combination of the CPs and anti-PD-1 treatment, tumor model mice were randomly assigned to four groups (10 mice per group) via random lottery: the PBS group, DETA-Dex group, anti-PD-1 antibody group, or combined DETA-Dex and anti-PD-1 antibody group. Mice were injected with 3 mg/kg DETA-Dex and 5 mg/kg anti-PD-1 antibody (clone RMP1-14, Bioxcell) via intratumoral injection. On Day 14, we measured tumor sizes with calipers and weighed the tumor samples upon harvest.

### Flow cytometry analysis

To prepare single-cell suspensions for flow cytometry, tumors were dissected into fragments and then digested with a protease cocktail containing collagenase IV (2 mg/ml) and DNase I (0.5 mg/ml) at 37 °C for 20 min and gently dissociated under a Miltenyi gentleMACS™ Dissociator and strained through 70 μm cell strainers. After red blood cell lysis, tumor-infiltrating leukocytes (TILs) were isolated using a tumor-infiltrating tissue leukocyte separation kit (WBC1092Z, TBD sciences, Tianjin, China) and blocked in 100 ml of 1% BSA for 30 min on ice.

Cells were incubated with FITC anti-mouse CD45 (#103,108, BioLegend, 2.5 μg/ml), BV421 anti-mouse CD4 (#100,438, BioLegend, 1.25 μg/ml), PE anti-mouse CD3 (#100,206, BioLegend, 2.5 μg/ml), APC anti-mouse CD8 (#100,712, BioLegend, 2.5 μg/ml), Brilliant Violet 711 anti-mouse CD3 (#100,241, BioLegend, 2.5 μg/ml), and APC anti-mouse CD25 (#102,012, BioLegend, 2.5 μg/ml). Cells were incubated with BV605 anti-mouse IFN-γ (#505,840, BioLegend, 2.5 μg/ml), BV711 anti-mouse IL-4 (#504,133, BioLegend, 5 μg/ml), and PE/Cyanine 7 anti-mouse IL17a (#506,922, BioLegend, 2.5 μg/ml) after using an Intracellular Staining Permeabilization Wash Buffer kit (#421,002, BioLegend). Cells were incubated with PE anti-mouse Foxp3 antibody (#320,008, BioLegend, 10 μg/ml) after using the True-Nuclear™ Transcription Factor Buffer kit (#424,401, BioLegend). Then, the cells were analyzed with a flow cytometer (Attune NxT device, Thermo Fisher Scientific). Examples of the gating strategy for flow cytometry analysis are shown in Additional file 1: Fig. S6. All antibodies and their isotype control antibodies were obtained from BioLegend (San Diego, CA, USA).

### PI staining and flow cytometry

The cells were stained with propidium iodide (PI; Beyotime Biotechnology) by modification of a protocol described previously [16]. 2.5 × 10^5^ 4T1 cells was seeded in 6-well plates and incubated at 37 °C under 5% CO_2_ for 24 h. For PI staining, 1 ml of CPs/RPMI1640 solution was added after removal of cell culture media followed by addition of 5 μl of 10 mg/ml PI/H_2_O solution. After the incubation, cells were trypsinized with trypsin—EDTA and centrifuged at 300 *g* for 5 min. Resulting cell pellets were then resuspended in PBS with 0.1% BSA and then the cells were analyzed with a flow cytometer.

### RNA isolation, qPCR, Western blot, and enzyme-linked immunosorbent assays

Total RNA from cells and tissues was extracted by using TRIzol reagent (Life Technologies). qPCR was performed by using ChamQ SYBR qPCR Master Mix (Vazyme Biotech Co., Ltd.) in a Step one™ Real-time PCR System (Applied Biosystems). Each sample was analyzed in triplicate and repeated in three independent assays. The level of each gene was normalized to that of GAPDH. The primers were synthesized by Sangon Biotech, and the primer sequences are shown in Additional file 1: Table S1.

Western blot analysis was performed according to the standard protocol. The protein samples were isolated with radioimmunoprecipitation assay (RIPA) buffer containing 1% protease inhibitor (Beyotime Biotechnology, Shanghai, China), and the total concentrations were detected using a bicinchoninic acid (BCA) protein assay kit (MicroBCA Kit, Thermo Scientific, USA). The proteins were mixed with SDS loading buffer and separated by sodium dodecyl sulfate–polyacrylamide gel electrophoresis (SDS–PAGE) and then transferred to polyvinylidene difluoride membranes (Bio-Rad, USA). The membranes were blocked with 5% skim milk and then incubated with primary antibodies (anti-TGFβ1, Ab ab179695, Abcam, USA; anti-β-actin, BM0627, Boster, China) overnight at 4 °C. The membranes were incubated with HRP-conjugated secondary antibodies (Life Technologies, USA) after three washes with PBST (PBS with 0.1% v/v Tween-20). After washing, the positive signals were visualized with fluorography using an enhanced chemiluminescence system (Cell Signaling Technology, USA).

Fresh tumor tissues were excised from the tumor-bearing mice 21 days after the tumors were established and then mechanically disrupted in PBS (0.1 g tissue/ml PBS) with EDTA-free protease inhibitor. Then, the tissues were homogenized in a Bead Beater apparatus (Tissuelyser-24, Jingxin Industrial Development Co., Ltd, Shanghai, China) with 5 mm beads for 30 s × 4 (60 Hz). The supernatants were separated by centrifugation (12,000 rpm, 4 °C for 20 min). The levels of the cytokines in the homogenates (TNFα, IL10, IL12, and TGFβ1) were then immediately measured with ELISA kits (eBioscience) following the manufacturer’s instructions.

### H&E staining and immunofluorescent staining

Tumors were processed as described and then fixed in 4% paraformaldehyde, embedded in paraffin, and sectioned into 5 μm sections. The slides were counterstained with hematoxylin and eosin (H&E) for histologic analyses. Rabbit anti-mouse TGFβ1 was first applied to the sections, followed by FITC-labeled donkey anti-rabbit secondary antibodies (KPL, Gaithersburg, MD, USA). Slides were imaged with a Zeiss LSM980 microscope with a Plan-Apochromat 10 × /0.45 objective.

### Statistical analysis

The results are expressed as the mean ± standard error of the mean (SEM). N refers to the number of animals per group. Differences between two groups were analyzed by two-tailed unpaired *t* test, and differences between groups were analyzed by one-way ANOVA with Dunnett’s tests. Two-way ANOVA with Dunnett’s multiple comparisons test and two-way ANOVA with Sidak’s multiple comparisons test were also used in this study. A value of *p* ≤ 0.05 was considered significant, ns, not significant. All statistical analyses were performed in GraphPad Prism 8.

## Supplementary Information

**Additional file 1: Figure S1.** Characterization of cDex and DETA-Dex. **Figure S2.** Kinetic analysis of the RNA droplets induced by the cationic polymers. **Figure S3.** Quantification of the transcription levels of common markers by RNA-seq. **Figure S4.** Gene Set Enrichment Analysis. **Figure S5.** Evaluation of antitumor activity of the cationic polymers in the BALB/c mouse model and BALB/c nude mouse model. **Figure S6.** Examples of the gating strategies for intracellular staining flow cytometry analysis. **Figure S7.** Evaluation of the antitumor activity of the cationic polymer combined with an anti-PD-1 antibody. **Table S1.** GPC analysis of the cationic polymers. **Table S2.** Dextran standards for GPC analysis. **Table S3.** qPCR primers and probes. **Table S4.** Flow cytometry antibodies.

**Additional file 2: Video S1.** The process of the intracellular formation and degradation of the PEI-RNA droplets.

**Additional file 3: Video S2.** The process of the intracellular formation and degradation of the cDex-RNA droplets.

**Additional file 4: Video S3.** The process of the intracellular formation and degradation of the DETA-Dex-RNA droplets.

## Abbreviations

LLPS: Liquid‒liquid phase separation
CPs: Cationic polymers
FRAP: Fluorescence recovery after photobleaching
RBPs: RNA-binding proteins
KEGG: Kyoto Encyclopedia of Genes and Genomes
FISH: Fluorescence in situ hybridization
GSEA: Gene set enrichment analysis
TME: Tumor microenvironment
