# Supplementary material for: Intracellular mRNA phase separation induced by cationic polymers for tumor immunotherapy
Source: J Nanobiotechnology. 2022 Oct 8;20:442. doi: 10.1186/s12951-022-01647-8 (PMC9548170; doi:10.1186/s12951-022-01647-8)
Supplement: Supplementary file 1 — Additional file 1: Figure S1. Characterization of cDex and DETA-Dex. Figure S2. Kinetic analysis of the RNA droplets induced by the cationic polymers. Figure S3. Quantification of the transcription levels of common markers by RNA-seq. Figure S4. Gene Set Enrichment Analysis. Figure S5. Evaluation of antitumor activity of the cationic polymers in the BALB/c mouse model and BALB/c nude mouse model. Figure S6. Examples of the gating strategies for intracellular staining flow cytometry analysis. Figure S7. Evaluation of the antitumor activity of the cationic polymer combined with an anti-PD-1 antibody. Table S1. GPC analysis of the cationic polymers. Table S2. Dextran standards for GPC analysis. Table S3. qPCR primers and probes. Table S4. Flow cytometry antibodies. [file 12951_2022_1647_MOESM1_ESM.docx]

Supplementary information

Intracellular mRNA phase separation induced by cationic polymers for tumor immunotherapy

Zhen Xing^1^, Jing Xue^1^, Xindian Ma^1^, Congwei Han^1^, Zhenzhen Wang^1^, Shunhuang Luo^1^, Chunming Wang^2^*, Lei Dong^1^*, Junfeng Zhang^1^*

^1^State Key Laboratory of Pharmaceutical Biotechnology, School of Life Sciences, Nanjing University, 163 Xianlin Avenue, Nanjing 210093, China.

^2^State Key Laboratory of Quality Research in Chinese Medicine, Institute of Chinese Medical Sciences, University of Macau, Taipa, Macau SAR 999078, China.

*Corresponding Authors: L.D., Email: [leidong@nju.edu.cn](mailto:leidong@nju.edu.cn); J.F.Z., Email: [jfzhang@nju.edu.cn](mailto:jfzhang@nju.edu.cn)

Table of Contents

[Figure S1 Characterization of cDex and DETA-Dex 3](#_Toc114778994)

[Figure S2 Kinetic analysis of the RNA droplets induced by the cationic polymers 4](#_Toc114778995)

[Figure S3 Quantification of the transcription levels of common markers by RNA-seq 5](#_Toc114778996)

[Figure S4 Gene Set Enrichment Analysis 7](#_Toc114778997)

[Figure S5 Evaluation of the antitumor activity of the cationic polymers in the BALB/c mouse model and BALB/c nude mouse model 8](#_Toc114778998)

[Figure S6 Examples of the gating strategies for intracellular staining flow cytometry analysis 9](#_Toc114778999)

[Figure S7 Evaluation of the antitumor activity of the cationic polymer combined with an anti-PD-1 antibody 10](#_Toc114779000)

[Table S1 GPC analysis of the cationic polymers 11](#_Toc114779001)

[Table S2 Dextran standards for GPC analysis 12](#_Toc114779002)

[Table S3 qPCR primers and probes 13](#_Toc114779003)

[Table S4 Flow cytometry antibodies 14](#_Toc114779004)

**Figure S1 Characterization of cDex and DETA-Dex**


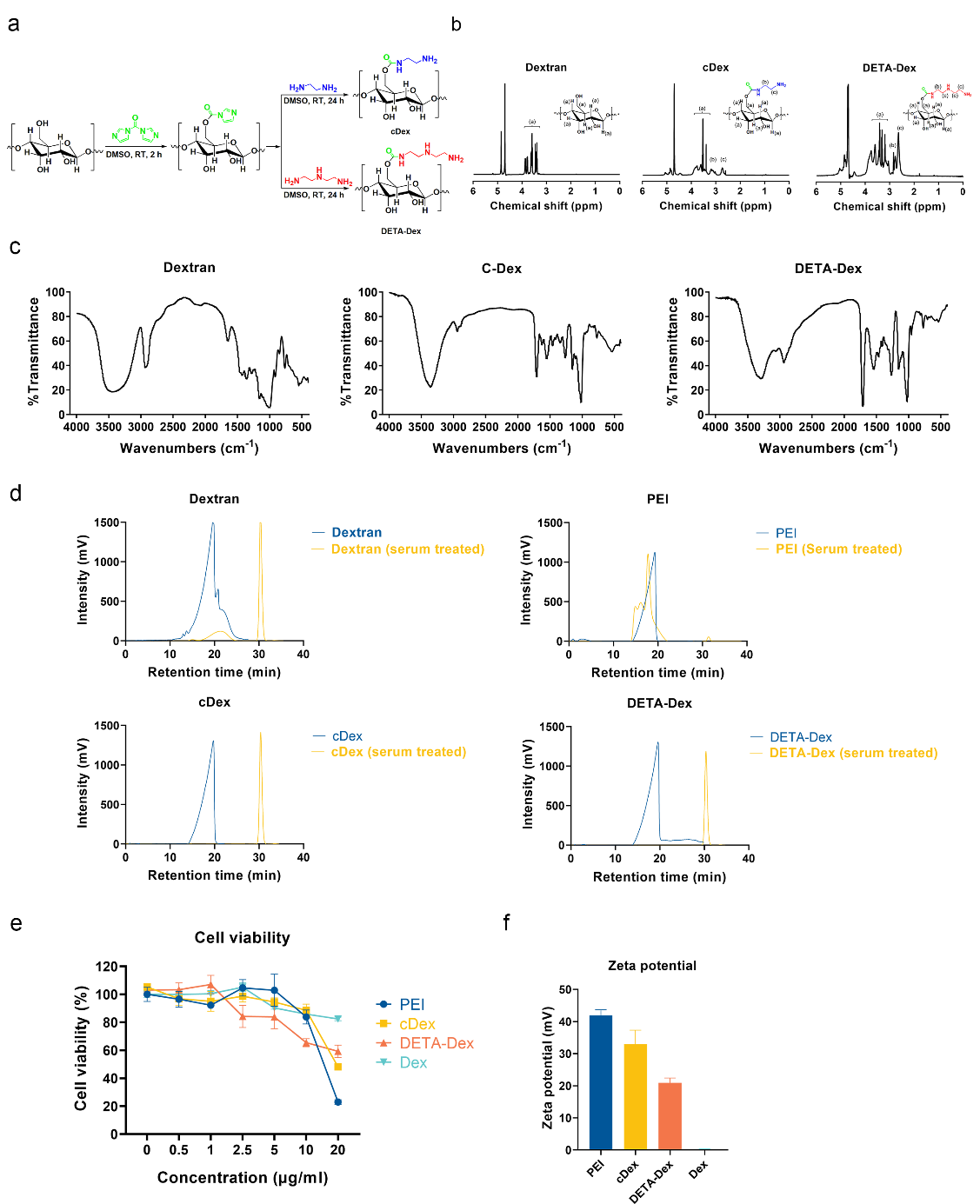


Fig. S1 Characterization of cDex and DETA-Dex. (a) Schematic illustration of cationic polymer synthesis. (b) ^1^H NMR and (c) FT-IR characterizations of the cationic polymers. (d) Evaluation of cationic polymer biodegradability. First, 50 mg of PEI, cDex or DETA-Dex were incubated with 50% fresh mouse serum for the indicated times. Then, the protein in each mixture was removed using the Sevag method, and the nondegraded cationic polymers and their degraded products were isolated and analyzed by GPC. (e) Cytotoxicity and (f) zeta potential characterizations of the cationic polymers.

**Figure S2** **Kinetic analysis of the RNA droplets induced by the cationic polymers**


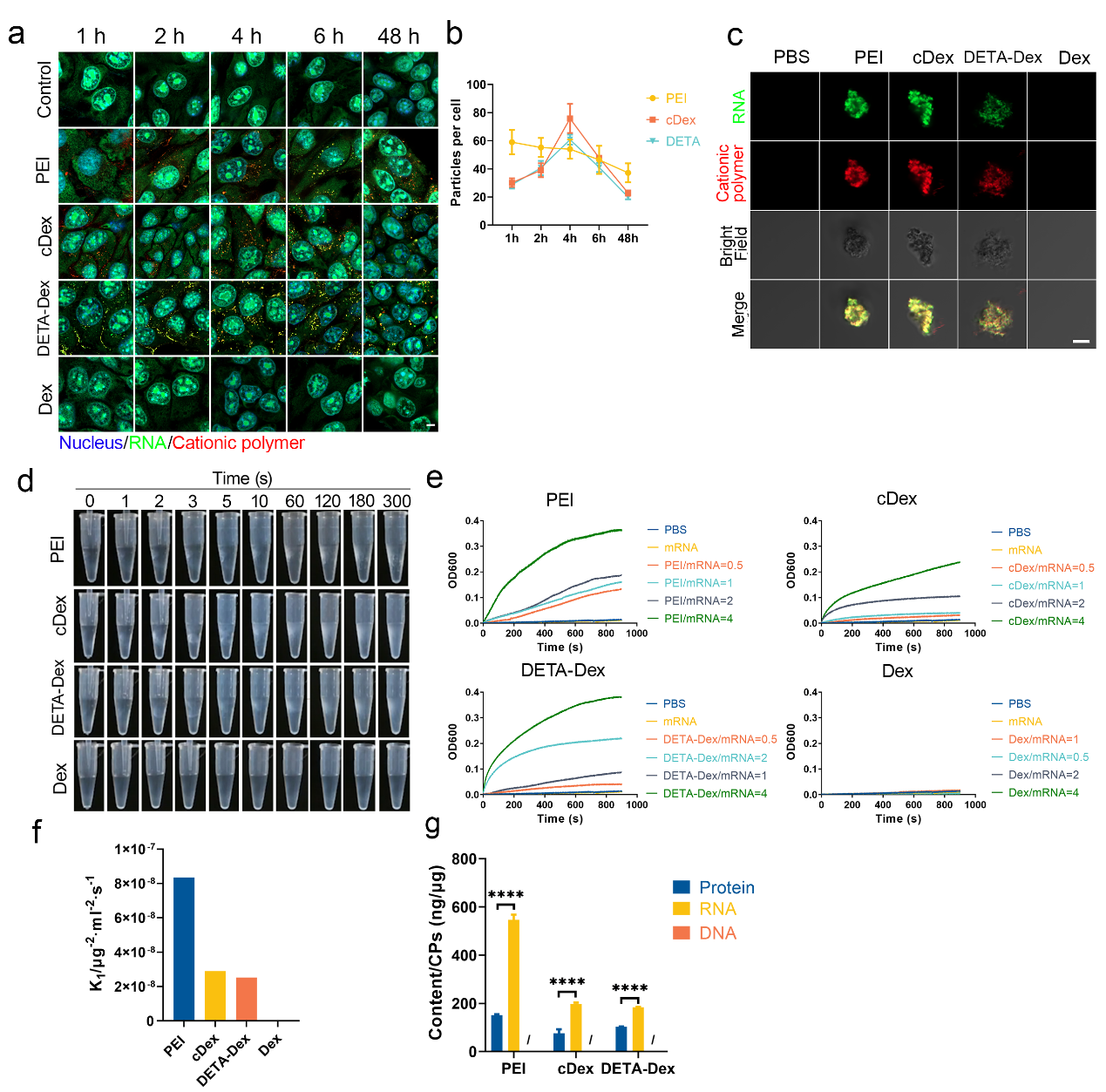


Fig. S2 Kinetic analysis of the RNA droplets induced by the cationic polymers. (a) Representative images of RNA droplet formation induced by the cationic polymers in 4T1 cells. Scale bar, 5 μm. (b) Quantification of the number of RNA droplets induced by the cationic polymers in 4T1 cells according to (a). (c) Confocal imaging of the suspension of RNA with cationic polymers in PBS. Scale bar, 5 μm. (d) Gross view of the RNA droplets induced by cationic polymers. First, 200 μg of 4T1 mRNA in 100 μl H_2_O was placed in a 0.2 ml [polypropylene PCR tube, then](https://ecatalog.corning.com/life-sciences/b2b/CN/en/Genomics-%26-Molecular-Biology/PCR-Consumables/PCR-Tubes-and-Strip-Tubes/Axygen%C2%AE-PCR-Strip-Tubes/p/PCR-0208-CP-C) 20 μg of cationic polymer was added. Videos were recorded with a digital camera. (e) Kinetic analysis of the turbidity (OD_600_) in PBS and RNA incubated with cationic polymers at various mass ratios (n = 3). (f) Kinetics constant of RNA droplet formation according to (e). (g) The contents of protein, RNA and DNA in the LLPS droplets (per μg of polymer) in 60 μl of cytoplasm incubated with 5 μg of PEI, cDex, or DETA-Dex.

**Figure S3** **Quantification of the transcription levels of common markers by RNA-seq**


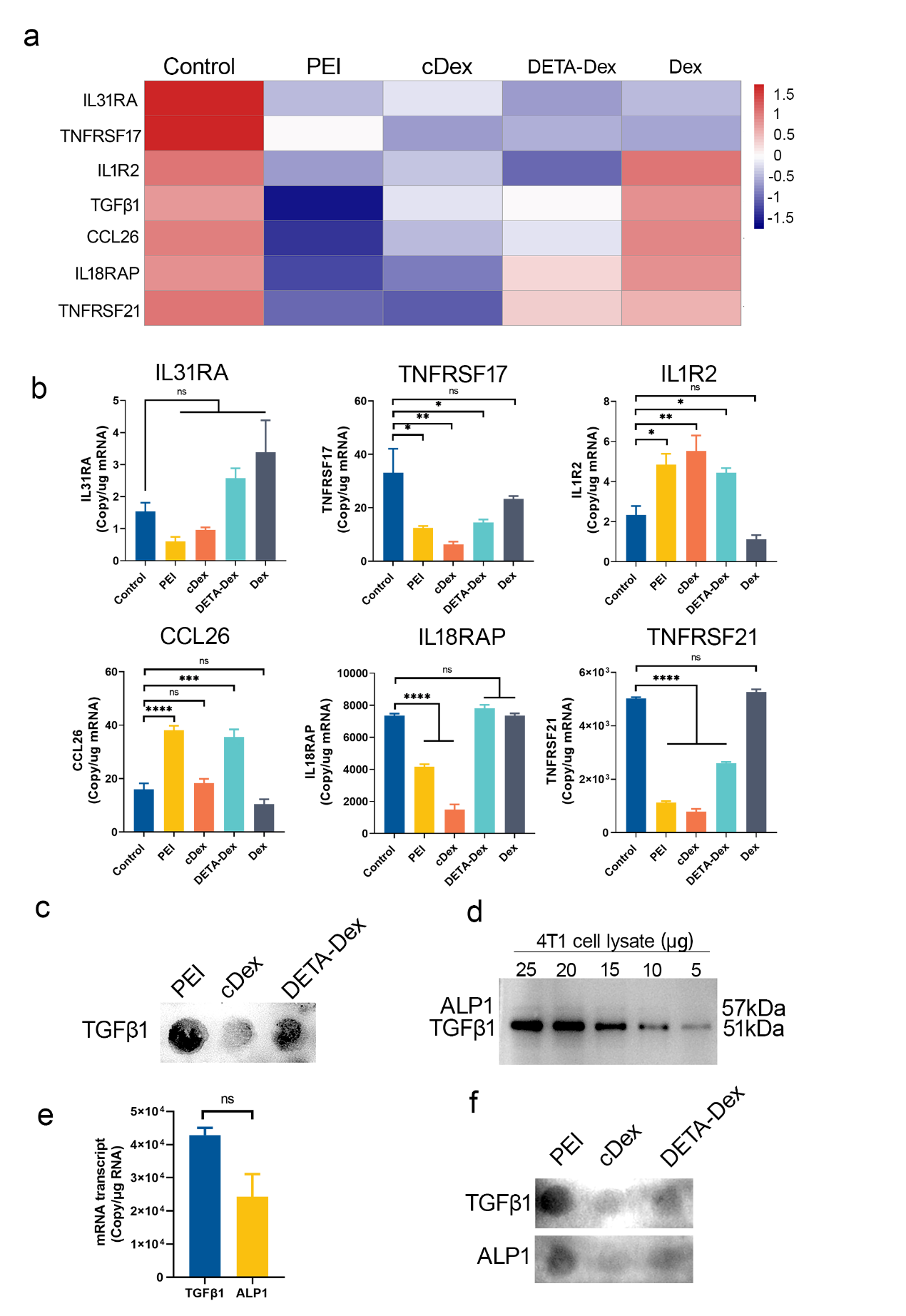


Fig. S3 Quantification of the transcription levels of common markers by RNA-seq. (a) Heatmap of common markers from the RNA-seq data. (b) Absolute quantification of the transcription levels of common markers by RNA-seq, including IL-1R2, IL31RA, CCL26, IL-18RAP, TNFRSF17 and TNFRSF21 after 6 h of CP treatment, in 4T1 cells. (c) Dot blot hybridization. The cationic polymers were incubated with mRNA from 4T1 cells for 30 min. The mRNA droplets were then centrifuged and spotted onto prepared nylon membranes. Then, the membranes were analyzed using a TGFβ1 DNA probe according to a standard northern blot protocol. (d) Western blot analysis of TGFβ1 and ALP1 in 4T1 cells. (e) Absolute quantification of the transcription levels of TGFβ1 and ALP1 in 4T1 cells. (f) Dot blot hybridization of TGFβ1 and ALP1 in the LLPS droplets formed by incubating 4T1 cytoplasm with PEI, cDex or DETA-Dex. Data are expressed as the mean ± SEM, and the differences between experimental groups were analyzed by one-way ANOVA with Dunnett’s test (b); ∗*p* < 0.05, ∗∗*p* < 0.01, ∗∗∗*p* < 0.001, ∗∗∗∗*p* < 0.0001.

**Figure S4 Gene Set Enrichment Analysis**


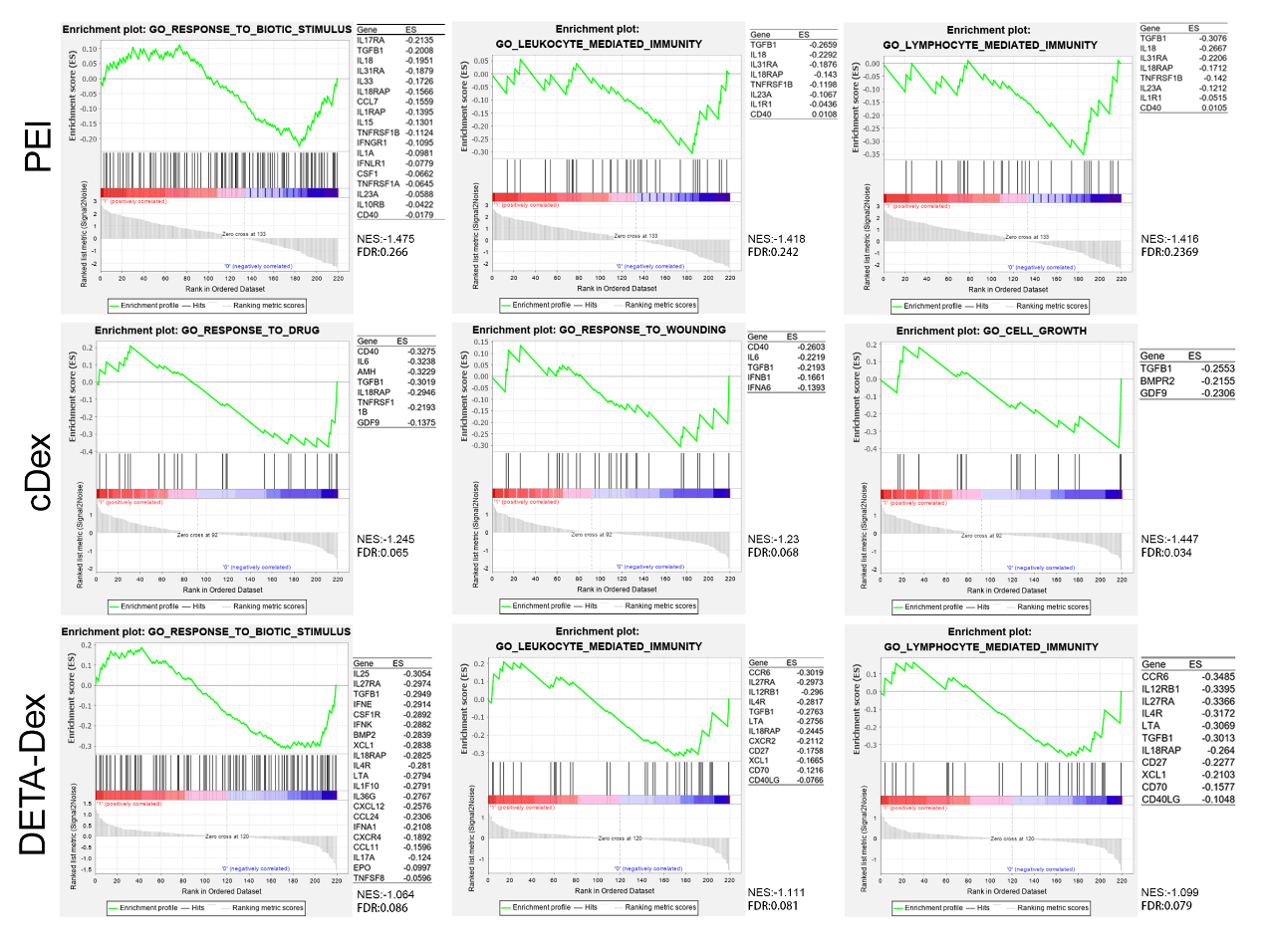


Fig. S4 GSEA highlighted a negative association with the ‘Go_Respone_to_Biotic_Stimulus’, ‘Go_Leukocyte_Mediated_Immunity’ and ‘Go_Lymphocyte_Mediated_Immunity signal’ pathways in the PEI and DETA-Dex groups and highlighted a negative association with the ‘Go_Response_To_Drug’, ‘Go_Response_To_Wounding’ and ‘Go_Cell_Growth’ pathways in the cDex group. The enrichment scores of the key proteins in the pathways are shown in the related tables. The enrichment scores were normalized for each gene set to account for the size of the set, yielding a normalized enrichment score (NES). The proportion of false-positive controls was calculated by determining the false discovery rate (FDR) corresponding to each NES.

**Figure S5** **Evaluation of the antitumor activity of the cationic polymers in the BALB/c mouse model and BALB/c nude mouse model**


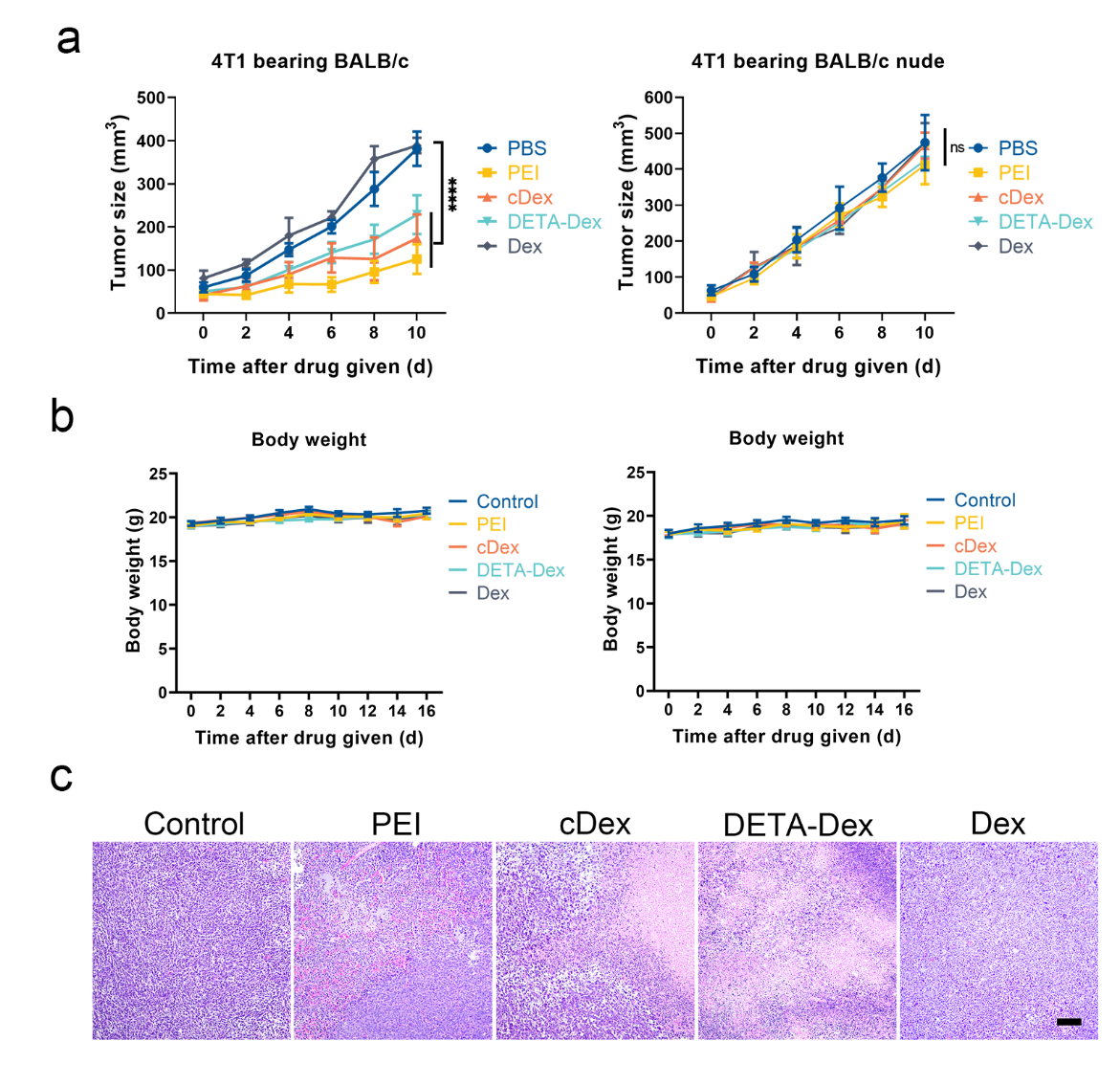


Fig. S5 Evaluation of the antitumor activity of the cationic polymers in the BALB/c mouse model and BALB/c nude mouse model. Female BALB/c and BALB/c nude mice were subcutaneously implanted with 1 × 10^6^ 4T1 cells. On Day 7, different CPs were administered to the mice *via* intratumoral injection (3 mg/kg body weight, dextran was used as a control reagent, n = 10 for each group). (a) Growth curves of the 4T1 tumors from the model animals treated with the cationic polymers. (b) Body weight curves of the animals treated with the cationic polymers. (c) Histological analysis (H&E staining) of the tumor sections from mice that received cationic polymer treatment (scale bar: 100 μm). (c) Data are expressed as the mean±SEM, and the differences between experimental groups were analyzed by two-way ANOVA with Sidak’s multiple comparisons test (A); ∗*p* < 0.05, ∗∗*p* < 0.01, ∗∗∗*p* < 0.001, ∗∗∗∗*p* < 0.0001.

**Figure S6 Examples of the gating strategies for intracellular staining flow cytometry analysis**


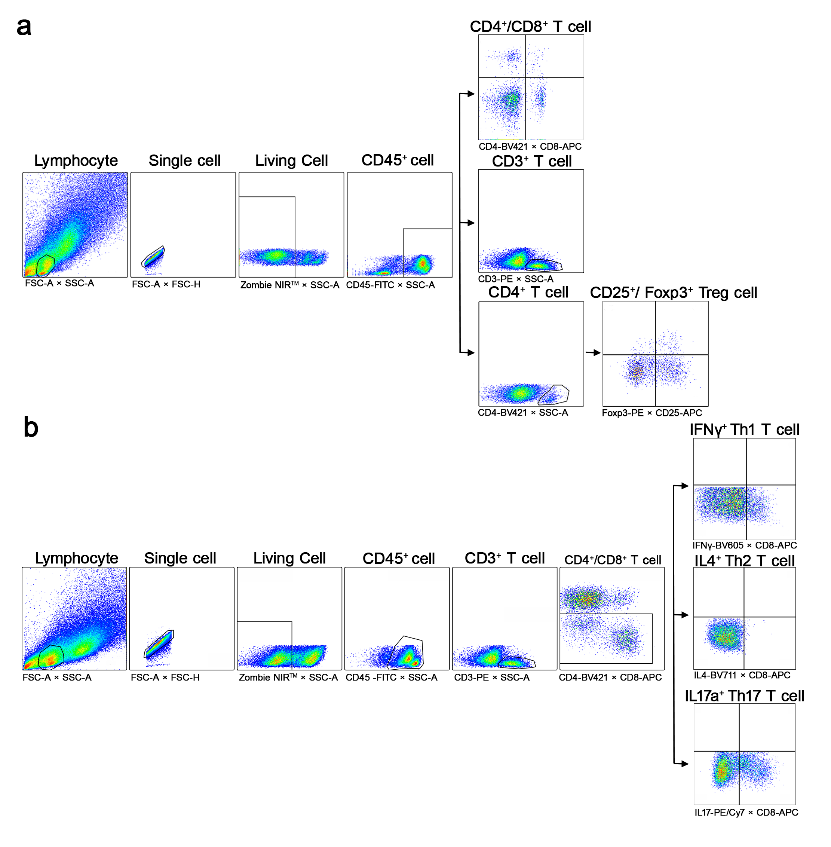


Fig. S6 Examples of the gating strategies for intracellular staining flow cytometry analysis. Related to Fig. 3h, Fig. 3i, Fig. 4f, Fig. 4g, Fig. 5g, and Fig. 5h.

**Figure S7 Evaluation of the antitumor activity of the cationic polymer combined with an anti-PD-1 antibody**


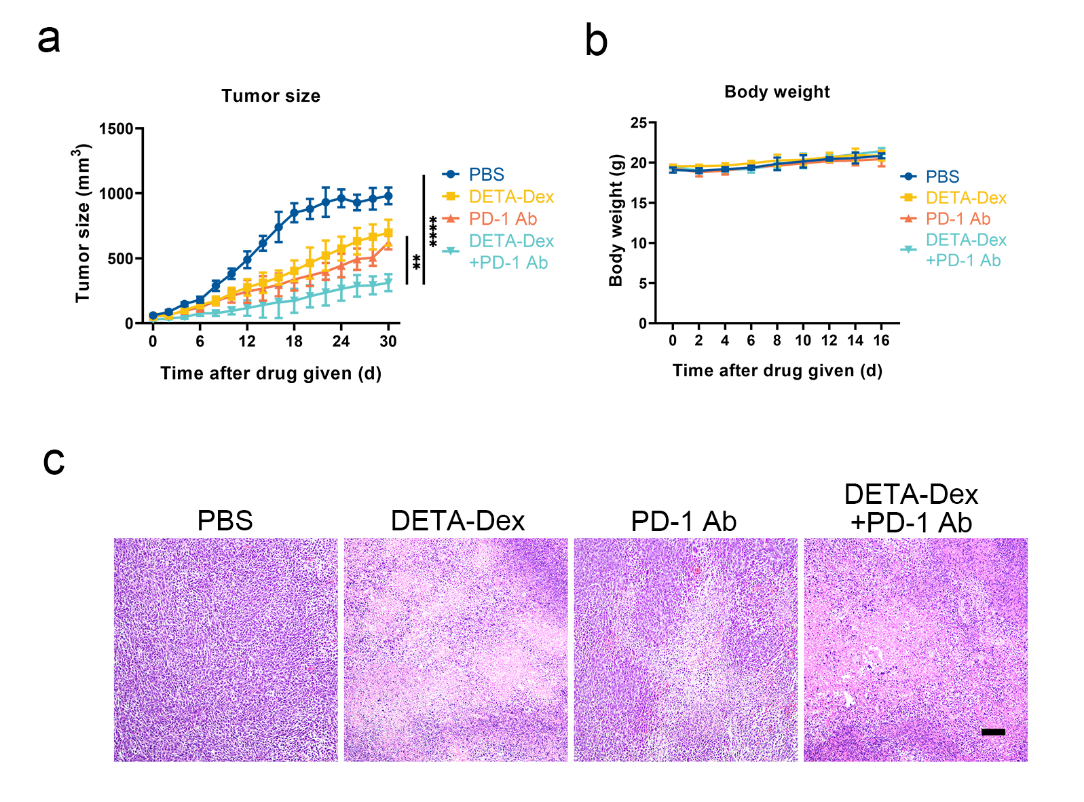


Figure S7 Evaluation of the antitumor activity of the cationic polymer combined with an anti-PD-1 antibody. Female BALB/c mice were subcutaneously implanted with 1 × 10^6^ 4T1 cells. On Day 7, the mice were injected with 3 mg/kg DETA-Dex or 5 mg/kg anti-PD-1 antibody *via* intratumoral administration (n = 10 for each group). (a) Growth curves of the 4T1 tumors from the model mice treated with DETA-Dex or anti-PD-1 antibody. (b) Body weight curves of the animals treated with DETA-Dex or anti-PD-1 antibody. (c) Histological analysis (H&E staining) of the tumor sections from mice that received cationic polymer or antibody treatment (scale bar: 100 μm). Data are expressed as the mean±SEM, and the differences between experimental groups were analyzed by two-way ANOVA with Sidak’s multiple comparisons test (A); ∗*p* < 0.05, ∗∗p < 0.01, ∗∗∗*p* < 0.001, ∗∗∗∗*p* < 0.0001.

**Table S1 GPC analysis of the cationic polymers**

|  | Dextran | cDex | DETA-Dex |
| --- | --- | --- | --- |
| Molecular weight/Mw | 105146.201 | 115981.534 | 11611.766 |
| Retention time/min | 19.797 | 19.631 | 19.696 |

**Table S2 Dextran standards for GPC analysis**

| Standard/Mw | 4000 | 11600 | 23900 | 50800 | 80900 | 275000 | 667800 |
| --- | --- | --- | --- | --- | --- | --- | --- |
| Retention time/min | 25.239 | 23.659 | 22.259 | 21.004 | 20.056 | 18.222 | 16.744 |

**Table S3 qPCR primers and probes**

| Name | Forward (5′‐3′) | Reverse (5′‐3′) |
| --- | --- | --- |
| mus-TGFβ1 | ATTCCTGGCGTTACCTTGGT | TGTATTCCGTCTCCTTGGTTCA |
| mus-IL1R2 | ATGAGCCAAGGATGTGGGTG | CCCAGAAACACTTTGCACGG |
| mus-IL18RAP | TTGTCCTGCTCTGTCGAACC | GGTCACATCTCGGTCAAGCA |
| mus-CCL26 | AGCAATTCTGTGTCCAGCCA | CACAAATGGTTCCTGGTGTTCA |
| mus-TNFRSF21 | ATGTTGACCGTACCACTGGC | CAGACAGTCCGTGTGAGCTT |
| mus-IL10 | ACATACTGCTAACCGACTCCTT | ACTCTTCACCTGCTCCACTG |
| mus-IL12a | CATCAACGCAGCACTTCAGA | CGCAGAGTCTCGCCATTATG |
| mus-β-actin | GACCTCTATGCCAACACAGTGC | GTACTCCTGCTTGCTGATCCAC |
| Mus-ALP1 | CTACCACTCGGGTGAACCAC | GCCATCTAGCCTTGTACCCC |
| TGFβ1-Probe 1 | TCATAGATGGCGTTGTTGCG |  |
| TGFβ1-Probe 2 | CCGAATGTCTGACGTATTGA |  |
| TGFβ1-Probe 3 | TAGAGTTCCACATGTTGCTC |  |
| ALP1-probe 1 | TTTTCAAGGTCTCTTGGGCTTGCTGTCGCC |  |
| ALP1-probe 2 | TGGCACAAAAGAGTTGGTAAGGCAGGTGCC |  |
| ALP1-probe 3 | CTGGCCCTTAAGGATTCGGGCAGCGGTTAC |  |

**Table S4 Flow cytometry antibodies**

| Name | Tag | Code | Isotype | Brand |
| --- | --- | --- | --- | --- |
| anti-mouse CD45 | FITC | # 103108 | Rat IgG2b, κ | BioLegend |
| anti-mouse CD3 | PE | # 100236 | Rat IgG2b, κ | BioLegend |
| anti-mouse CD3 | BV711 | # 100241 | Rat IgG2b, κ | BioLegend |
| anti-mouse CD4 | BV421 | # 100443 | Rat IgG2b, κ | BioLegend |
| Anti-mouse CD8a | APC | # 100712 | Rat IgG2a, κ | BioLegend |
| anti-mouse CD25 | APC | # 101910 | Rat IgG2b, κ | BioLegend |
| anti-mouse Foxp3 | PE | # 126404 | Rat IgG2b, κ | BioLegend |
| anti-mouse IFN-γ | BV605 | # 505840 | Rat IgG1, κ | BioLegend |
| anti-mouse IL4 | BV711 | # 504133 | Rat IgG1, κ | BioLegend |
| anti-mouse IL17a | PE-Cy7 | # 506922 | Rat IgG1, κ | BioLegend |
| Rat IgG2b, κ Isotype Ctrl | FITC | # 400606 | Rat IgG2b, κ | BioLegend |
| APC Rat IgG2b, κ Isotype Ctrl | APC | # 400612 | Rat IgG2b, κ | BioLegend |
| BV421 Rat IgG2b, κ Isotype Ctrl | BV421 | # 400655 | Rat IgG2b, κ | BioLegend |
| PE Rat IgG2b, κ Isotype Ctrl | PE | # 400608 | Rat IgG2b, κ | BioLegend |
| BV605 Rat IgG1, κ Isotype Ctrl | BV605 | # 400434 | Rat IgG1, κ | BioLegend |
| BV711 Rat IgG1, κ Isotype Ctrl | BV711 | # 400441 | Rat IgG1, κ | BioLegend |
| PE-Cy7 Rat IgG1, κ Isotype Ctrl | PE-Cy7 | # 400415 | Rat IgG1, κ | BioLegend |
| Zombie NIR™ Fixable Viability Kit | - | # 423105 | - | BioLegend |
